# Investigating the Composition and Metabolic Potential of Microbial Communities in Chocolate Pots Hot Springs

**DOI:** 10.3389/fmicb.2018.02075

**Published:** 2018-09-07

**Authors:** Nathaniel W. Fortney, Shaomei He, Brandon J. Converse, Eric S. Boyd, Eric E. Roden

**Affiliations:** ^1^Department of Geoscience, NASA Astrobiology Institute, University of Wisconsin-Madison, Madison, WI, United States; ^2^Department of Bacteriology, University of Wisconsin-Madison, Madison, WI, United States; ^3^Department of Microbiology and Immunology, NASA Astrobiology Institute, Montana State University, Bozeman, MT, United States

**Keywords:** chocolate pots, yellowstone, iron(III)-reducing bacteria, iron(II)-oxidizing bacteria, metagenomics, extracellular electron transfer

## Abstract

Iron (Fe) redox-based metabolisms likely supported life on early Earth and may support life on other Fe-rich rocky planets such as Mars. Modern systems that support active Fe redox cycling such as Chocolate Pots (CP) hot springs provide insight into how life could have functioned in such environments. Previous research demonstrated that Fe- and Si-rich and slightly acidic to circumneutral-pH springs at CP host active dissimilatory Fe(III) reducing microorganisms. However, the abundance and distribution of Fe(III)-reducing communities at CP is not well-understood, especially as they exist *in situ*. In addition, the potential for direct Fe(II) oxidation by lithotrophs in CP springs is understudied, in particular when compared to indirect oxidation promoted by oxygen producing Cyanobacteria. Here, a culture-independent approach, including 16S rRNA gene amplicon and shotgun metagenomic sequencing, was used to determine the distribution of putative Fe cycling microorganisms in vent fluids and sediment cores collected along the outflow channel of CP. Metagenome-assembled genomes (MAGs) of organisms native to sediment and planktonic microbial communities were screened for extracellular electron transfer (EET) systems putatively involved in Fe redox cycling and for CO_2_ fixation pathways. Abundant MAGs containing putative EET systems were identified as part of the sediment community at locations where Fe(III) reduction activity has previously been documented. MAGs encoding both putative EET systems and CO_2_ fixation pathways, inferred to be FeOB, were also present, but were less abundant components of the communities. These results suggest that the majority of the Fe(III) oxides that support *in situ* Fe(III) reduction are derived from abiotic oxidation. This study provides new insights into the interplay between Fe redox cycling and CO_2_ fixation in sustaining chemotrophic communities in CP with attendant implications for other neutral-pH hot springs.

## Introduction

Environments containing high concentrations of redox active elements, such as iron (Fe), are important areas of study because of the potential for these elements support the energy metabolism of microbial cells. In its oxidized state [Fe(III)] Fe can serve as a terminal electron acceptor for dissimilatory iron reduction (DIR) by Fe(III)-reducing bacteria (FeRB) (Lovley et al., 2004). In its reduced form [Fe(II)] Fe can serve as an electron donor for lithoautotrophic Fe(II)-oxidizing bacteria (FeOB) (Emerson et al., 2010). Although less prominent in modern Earth environments, Fe(II) can also serve as an electron donor for photosynthetic reactions (Crowe et al., 2008; Llirós et al., 2015; Camacho et al., 2017). Fe is the most abundant redox-active metal in the Earth's crust (Taylor and McLennan, 1985) and on astrobiologically relevant worlds, like Mars (Taylor and McLennan, 1985, 2009). Researchers have suggested that both Fe(II) oxidation and Fe(III) reduction have been active microbial metabolic processes since before the Great Oxidation Event (ca. 2.4 Ga) when Fe(II) concentrations in the Archean ocean were high (Hafenbradl et al., 1996; Vargas et al., 1998; Emerson, 2000). Additionally, it is hypothesized that DIR was one of the earliest forms of microbial respiration (Vargas et al., 1998).

Chocolate Pots (CP) is an Fe(II)- and Si-rich circumneutral-pH geothermal spring in the northwestern portion of Yellowstone National Park. The anoxic spring water issuing from the vent source at CP is of a similar composition to what is predicted for the Archean ocean (Canfield, 2005). Additionally, mineralogical analyses of the Martian surface have identified deposits indicative of circumneutral-pH (Arvidson et al., 2014), and relic hot spring environments (Squyres et al., 2008; Ruff and Farmer, 2016). Together, this makes CP a suitable analog environment in terms of gaining insight into metabolic processes that could have supported life on early Earth and possibly Mars.

For the past two decades, investigators have used CP as a model environment to study ancient Fe deposition by focusing on the role the microbial community plays in the formation of Fe oxide deposits. In particular, significant attention has been placed on understanding the role of oxygen produced by photosynthetic microbial mat communities in promoting the indirect, abiotic oxidation of Fe(II) (Pierson et al., 1999; Pierson and Parenteau, 2000; Trouwborst et al., 2007; Parenteau and Cady, 2010; Parenteau et al., 2014). The potential for lithoautotrophic Fe(II) oxidation has been considered as well, however after unsuccessful culturing of putative FeOB (Emerson and Weiss, 2004) and little experimental evidence to support their activity in the microbial mats (Trouwborst et al., 2007), research has not been continued in this area.

The potential for DIR in redox transformation of Fe-Si oxides at CP was cited early on (Pierson et al., 1999), but in-depth studies of the anaerobic heterotrophic microbial community have been relatively recent. For example, natural amorphous Fe(III)-oxides from CP were shown to host communities containing known and putative FeRB (Fortney et al., 2016). Subsequent incubation experiments combined with stable isotope probing (SIP) experiments using ^13^C-labeled acetate identified putative FeRB under acetate-stimulated and unamended incubation conditions (Fortney et al., 2018). However, constraints on the spatial distribution of FeRB within the sediment column of CP were not examined in detail.

In this study we used DNA sequencing to further investigate the spatial distribution of microorganisms involved in Fe cycling in CP vent waters, along the flow path of the outflow channel, and as a function of sediment column depth along the flow path. 16S rRNA gene amplicon sequencing was conducted on filtered spring water samples and sediment core samples collected from the vent and further downstream with increasing distances from the vent pool. Shotgun metagenomic sequences were obtained from the top 1 cm of three of these sediment cores as well as filtered vent pool water biomass in order to identify abundant taxa containing genes involved in extracellular electron transfer (EET) and CO_2_ fixation. Our results provide further support for an active FeRB community in the CP sediments, especially proximal to the vent pool. In contrast, although our genomic data supports the metabolic potential for lithoautotrophic FeOB, they do not appear to be prominent members of the microbial community.

## Materials and methods

### Sample collection and processing

**Figure 1:**
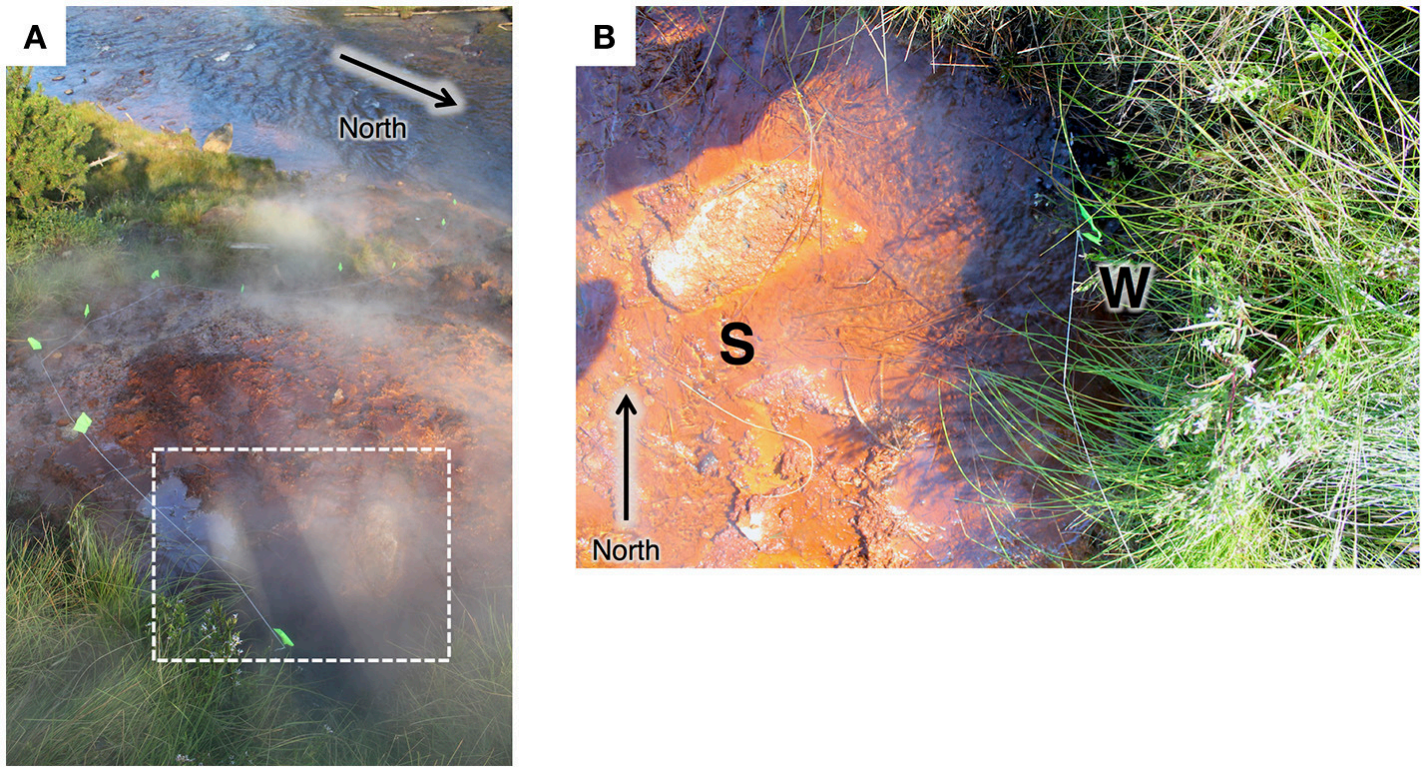
**(A)** View from the top of the main hot spring mound looking toward the Gibbon River. Flow path is marked with neon-green flags. Approximate area of the vent pool is marked with a white dotted line. **(B)** Top-down view of the pool at the main hot spring vent pool at Chocolate Pots. The site for the sediment core collection in 2013 is indicated with an S. The vent source (partially obscured by grasses) where spring water was collected in 2015 is indicated with a W.

A total of six small (ca. 1 × 10 cm) sediment cores were collected from the CP vent pool and along the flow path in August 2013 (Figure 1). Spring water was filtered from the hot spring source and the vent pool source in October 2015 using an in-line 0.2 μm polyethersulfone (PES) membrane and a peristaltic pump. In an anaerobic chamber, core samples were thawed, extruded, and sectioned into 1 cm intervals. Subsections were split for sequential HCl extraction for Fe geochemical and isotope analyses (Fortney et al., in prep.) and DNA extraction.

### DNA extraction and sequencing

DNA was extracted according to previously described methods (Fortney et al., 2016). DNA extracts from the core samples were PCR amplified using the universal primer set 515f/806r (Caporaso et al., 2011) targeting 16S rRNA genes and were multiplexed using standard Roche MID primer tags. Amplicons were sequenced at the University of Wisconsin Biotech Center (UWBC, https://www.biotech.wisc.edu/) using the Roche 454 FLX+ pyrosequencing platform. DNA from the top 1 cm sample from cores 1, 2, and 3 was submitted to UWBC for paired-end 2 × 100 bp Illumina HiSeq 2000 shotgun metagenomic sequencing.

PES membrane filters were cut in half and sliced into strips using a sterile razor blade for use in DNA extraction. DNA from replicate extracts was pooled and submitted to UWBC for paired-end 2 × 250 bp Illumina HiSeq Rapid shotgun metagenomic sequencing. An additional DNA sample was submitted to Argonne National Labs for PCR amplification (using the universal primers 515f/806r) prior to paired-end 2 × 200 bp Illumina MiSeq 16S amplicon sequencing.

### Analysis of 16S rRNA gene amplicon data

Raw sequences were processed using QIIME following the protocol for 454 pyrosequencing data or Illumina MiSeq 16S rRNA gene amplicon sequencing data, according to previously published methods (Fortney et al., 2016, 2018).

### Metagenome assembly, binning, and assessment of MAGs

Raw shotgun metagenomic sequence from the CP sediment core DNA samples was assembled using metaSPAdes 3.9 (Bankevich et al., 2012; Nurk et al., 2016). Binning was accomplished using CONCOCT 0.4.0 (Alneberg et al., 2014) along with some manual binning based on %GC and coverage to produce metagenome-assembled genomes (MAGs). Differential read coverage was obtained by mapping reads from each metagenome against the contigs from the combined metagenomic assembly (co-assembly) using SNAP 0.15.4 (Zaharia et al., 2011) with default settings, and Samtools 1.3.1 to obtain the coverage of each contig (http://samtools.sourceforge.net) (Li et al., 2009). MAG quality (i.e., completeness, contamination, and strain heterogeneity) was determined using CheckM 1.0.7 (Parks et al., 2015). Putative phylogenetic identities of each MAG were determined through a consensus between the identities provided by CheckM and the classification based on the lowest common ancestor of essential housekeeping genes based on sequence homology. The CheckM algorithm infers phylogeny based on placement of the MAG within the reference genome tree constructed from 43 conserved phylogenetic marker genes. The 111 bacterial housekeeping genes expected to be encoded in each MAG were identified using previously described methods (Albertsen et al., 2013), including gene prediction by Prodigal (Hyatt et al., 2010) and essential housekeeping gene identification by HMMer search against HMM models (Finn et al., 2011); protein sequences of the detected essential housekeeping genes were aligned to the NCBI nr database (current as of June 8, 2016) using BLASTp. BLAST output was input into MEGAN (Huson et al., 2007) to determine the lowest common ancestor of these genes to aid in taxonomic classification of each MAG. Dendroscope 3.5.9 (Huson and Scornavacca, 2012) was used to project phylogenetic trees using the CheckM output.

Metagenomic sequence data from the vent pool DNA sample was processed identically with the following exceptions: Raw reads were quality-trimmed, merged, and sequencing adapters were removed in CLC Genomic Workbench 7.5.1 (http://www.clcbio.com) at the UWBC computer center. Processed reads were assembled with raw reads in metaSPAdes 3.10 using the “trusted contigs” command in order to improve assembly quality (e.g., N50). Manual kmer sizes of 21, 33, 55, 77, 99, and 127 were used for assembly. Read mapping was unnecessary in the Vent metagenome because it was a single sample, and coverage for each contig is contained in the metaSPAdes output. Assembly, automated binning, read mapping, and BLAST for both the CP core and vent pool metagenomes were all run using the UW-Madison Center For High Throughput Computing (CHTC) in the Department of Computer Sciences (http://chtc.cs.wisc.edu/).

### Inference of metabolic potential

Metagenomic assemblies were uploaded to IMG/M ER (https://img.jgi.doe.gov/cgi-bin/mer/main.cgi) for gene annotation (Mavromatis et al., 2009). Metagenomes were screened for homologs of EET systems found in FeRB (e.g., the *Geobacter*-like *pcc* system; Liu et al., 2014; Shi et al., 2014) using previously published methods (Fortney et al., 2016). Metagenomes were also screened for EET systems found in known FeRB (*Shewanella* spp.*, mtrABC*; Hartshorne et al., 2009) and FeOB (*Acidithiobacillus ferrooxidans, cyc2*; *Sideroxydans* spp. *mtoABCD*; *Rhodobacter ferrooxidans, foxEZY*; and *Rhodopseudomonas palustris, pioABC*; Ilbert and Bonnefoy, 2013, and references therein) using command-line BLAST and the BLASTp function in IMG. Genes coding for putative EET systems, which are not homologous to known models, were identified according to previously published methods (Fortney et al., 2018). MAGs encoding putative outer-membrane porins, and multiheme *c-*type cytochromes (*c-*cyts) with predicted extracellular and periplasmic locations, as well as other supplemental genes predicted to be involved in Fe transformation pathways, are hereafter referred to as *pcc*-like EET systems. MAGs with genes fitting the above criteria but lacking extracellular *c-*cyts are hereafter referred to as *mto*-like EET systems.

Metagenomes were screened for four different CO_2_ fixation pathways: the reductive pentose phosphate cycle [Calvin-Benson-Bassham (CBB)], reductive tricarboxylic acid cycle (rTCA), reductive acetyl-CoA pathway [Wood-Ljungdahl (WL)], and 3-hydroxypropionate (3HP) bicycle. Metagenomes were not screened for the 3-hydroxypropionate/4-hydroxybutyrate pathway or the dicarboxylate/4-hydroxybutyrate pathway because these systems are thus far restricted to thermophilic Archaea isolated from hydrothermal systems much hotter than CP (Hügler and Sievert, 2011). MAGs potentially involved in CO_2_ fixation were positively identified by the presence of all genes predicted to be in a given pathway. Details are provided in Supplementary Material 1.1.

To determine whether CO_2_ fixation pathways identified were associated with lithotrophs or phototrophs, MAGs related to known phototrophic organisms were screened for phototrophy-related genes. Firstly, MAGs were screened for genes coding for photoreaction centers and associated photosynthetic genes [e.g., photosystems II and I (PS-II and -I), *puhA* and *pufLM*] using queries from anoxygenic (*Chloroflexus aurantiacus* J-10-fl, *R. palustris* 42OL, *Blastochloris viridis* DSM 133, and *Roseiflexus castenholzii* HLO8) and oxygenic (*Cyanothece* sp. BH68, *Oscillatoria* sp. PCC 10802, *Pseudanabaena* sp. PCC 6802, and *Synechococcus* sp. JA-3-3Ab) phototrophs within the IMG database. Next, MAGs were screened for photosynthetic gene categories in annotations (e.g., pfam, COG, EC).

### Linking 16S rRNA amplicon data to MAGs

16S rRNA gene sequences were recovered from the metagenomic libraries using the CheckM algorithm and aligned to the respective 16S rRNA gene in the amplicon libraries by BLASTn search. This allowed for identification of a representative MAG for a given 16S rRNA sequence defined OTU, and vise-versa in order to correlate abundant MAGs and OTUs between sequence libraries.

### Accession numbers and sequence files

All metagenomic contigs for the CP core metagenomic co-assemblies and the CP vent pool water column assembly are available through IMG/M ER under taxon identification numbers 3300010938 and 3300014149, respectively. Nucleotide and amino acid sequences for each of the MAGs from the CP core metagenomic co-assembly and the CP vent pool water column assembly are available as compressed FASTA files in the Supplemental Material of this paper. Processed reads (FASTA files) from the 16S rRNA gene amplicon sequencing of the CP cores and CP vent pool water column, and raw OTU table text files are available as in the Supplementary Material of this paper.

## Results and discussion

### Description of chocolate pots hot springs

The Chocolate Pots are a series of vent features along and within the Gibbon River ~5 km south of the Norris Geyser Basin (Allen and Day, 1935; McCleskey et al., 2010). The hot spring studied here (Thermal ID: GCPNN002; 44.71008,−110.7413) is located along the southeastern bank of the Gibbon River and is comprised of a main hot spring vent and pool which flows over Fe(III) oxide deposits about 10 m down the bank to the river. The vent pool and flow path (see Figure 1) were the foci of this study. Two satellite vents located about half way down the bank were not sampled as part of this study.

The temperature of the core sampling site in the vent pool in 2013 was 50.7°C, and decreased to 40.8°C at the collection site of core 6. The temperature where the effluent from CP meets the Gibbon River was 38.1°C. The pH of the Vent coring site was 5.94, increasing to 7.90 at the site of core 6, and 8.25 upon entering the river. The concentration of aqueous Fe(II) was ca. 0.1 mmol L^−1^ at the Vent and decreased to <0.01 mmol L^−1^ by the site of core 4. Water was at a slightly higher temperature (ca. 51.4°C), and lower pH (ca. 5.79) at the vent source where water samples were collected in 2015 (see Supplementary Table 1 for details).

### Composition of the CP sediment cores and vent pool microbial communities: 16S rRNA gene amplicon sequence analysis

#### CP sediment cores

A total of 370544 high-quality 16S rRNA gene amplicon sequences were obtained from 42 sediment core subsamples. Following quality trimming and processing through QIIME (e.g., OTU picking) a total of 18088 reads were distributed between 885 OTUs (excluding singletons) at 97% identity. Overall, the microbial community of the CP core samples is diverse with only 22 OTUs (out of 320 OTUs collapsed to the Family level) having >1% read abundance in the 16S amplicon library (Table 1). However, these few OTUs comprise 61% of all reads in the libraries. OTUs with unassigned taxa comprised 10–15% of the reads.

**Table 1:** Microbial community composition of Chocolate Pots core samples.

**Sample**	**Greengenes taxonomic assignment^a^**	**% Total^b^**	**Representative species match^d^**	**Accession number**	**Identity (%)**	**Inferred physiology**	**References**	**Representative MAG^e^**	**Similarity (%)**
Combined core libraries (*n* = 42)	Crenarchaeota (p)	9.0	Anaerobic methanogenic archaeon ET1-8	AJ244284.1	87	Methanogenic cellulolytic archaeon	Chin et al., 1999	N/A^f^	
	Chlorobi (p)	5.0	*Ignavibacterium album* strain JCM 16511	NR_074698.1	83	Facultative anaerobic heterotroph	Iino et al., 2008; Liu et al., 2012	39	99.3
	Chlorobi (p)	4.7	*I. album* strain JCM 16511	NR_074698.1	84	Facultative anaerobic heterotroph	Iino et al., 2008; Liu et al., 2012	N/A	
	*Acetothermales* (o)	4.7	“*Candidatus* Acetothermus autotrophicum”	AP011801.1	83	Lithoautotrophic acetogen	Nunoura et al., 2005; Takami et al., 2012	N/A	
	*Nitrospirales* (o)	3.8	Uncultured bacterium clone Fl-1F_E11	EF220517.1	92	Uncharacterized	Yergeau et al., 2007	N/A	
	GAL15 (p)	3.6	*Thermovenabulum ferriorganovorum* strain Z-9801	NR_042719.1	86	Fe(III) reducer	Zavarzina et al., 2002	N/A	
	Acidobacteria (p)	3.5	*Thermanaerovibrio* sp. R101	FN556061.1	87	Uncharacterized thermophile	Sayeh et al., unpublished data	N/A	
	Crenarchaeota (p)	3.1	Anaerobic methanogenic archaeon ET1-8	AJ244284.1	91	Methanogenic cellulolytic archaeon	Chin et al., 1999	N/A	
	*Anaerolineae* (c)	2.5	Chloroflexi bacterium SCGC AAA240-O05	HQ675640.1	88	Uncharacterized	Swan et al., 2011	N/A	
	*Cenarchaeaceae* (f)	2.2	“*Ca*. Nitrosotenuis sp. AQ6f”	CP024808.1	97	Ammonia oxidizer	Sauder et al., unpublished data	N/A	
	*Thaumarchaeota* (c)	2.1	*Nitrososphaera viennensis* strain EN76	NR_134097.1	93	Ammonia oxidizer	Stieglmeier et al., 2014	N/A	
	*Syntrophobacteraceae* (f)	2.1	*Thermodesulforhabdus* sp. nov. M40/2 CIV-3.2	AF170420.1	87	Thermophilic sulfate oxidizer	Beeder et al., 1995; Sievert and Kuever, 2000	N/A	
	NC10 (p)	2.1	“*Ca*. Methylomirabilis oxyfera”	FP565575.1	89	Methane oxidizer, nitrate reducer	Ettwig et al., 2010	N/A	
	*Methylomirabiliaceae* (f)	1.9	“*Ca*. M. oxyfera”	FP565575.1	93	Methane oxidizer, nitrate reducer	Ettwig et al., 2010	N/A	
	Chloroflexi (p)	1.7	Chloroflexi bacterium SCGC AAA240-C09	HQ675555.1	88	Uncharacterized	Swan et al., 2011	N/A	
	*Aigarchaeota* (c)	1.7	“*Ca*. Caldiarchaeum subterraneum”	AP011878.1	85	Chemolithotroph	Nunoura et al., 2011	N/A	
	*Betaproteobacteria* (c)	1.4	*Sideroxydans paludicola* strain BrT	DQ386858.1	98	Lithoautotrophic Fe(II) oxidizer	Weiss et al., 2007	N/A	
	SBR1093 (p)	1.4	Bacterium Kaz2	AB491166.1	90	Uncharacterized	Sueoka et al., unpublished data	N/A	
	NC10 (p)	1.3	“*Ca*. Methylomirabilis sp. RS3”	KU891932.1	88	Denitrifying methanotroph	He and Hu, unpublished data	N/A	
	*Methanomassiliicoccaceae* (f)	1.2	*Methanomassiliicoccus* sp. N89-2	LN827539.1	92	Methanogen	Huynh et al., 2016	1	99.3
	*Acetothermaceae* (f)	1.0	“*Ca*. A. autotrophicum”	AP011801.1	97	Lithoautotrophic acetogen	Nunoura et al., 2005; Takami et al., 2012	N/A	
	Acidobacteria (p)	1.0	Bacterium Ellin7505	HM748715.1	85	Uncharacterized	Davis et al., 2011	N/A	
		61.2^c^							
Core 1 surface	Crenarchaeota (p)	8.5	Anaerobic methanogenic archaeon ET1-8	AJ244284.1	87	Methanogenic cellulolytic archaeon	Chin et al., 1999	N/A	
	*Thermodesulfovibrionaceae* (f)	8.1	*Thermodesulfovibrio yellowstonii* DSM 11347	CP001147.1	90	Fe(III), sulfate reduction	Henry et al., 1994; Sekiguchi et al., 2008	N/A	
	*Anaerolineae* (c)	7.3	*Dehalococcoides* sp. BHI80-15	AJ431246.1	87	Uncharacterized thermophile	Cambon-Bonavita et al., unpublished data	N/A	
	Armatimonadetes (p)	6.0	*Dictyoglomus turgidum* DSM 6724	NR_043385.1	88	Uncharacterized thermophile	Lucas et al., unpublished data	N/A	
	*Anaerolineae* (c)	5.3	Chloroflexi bacterium SCGC AAA240-O05	HQ675640.1	88	Uncharacterized	Swan et al., 2011	N/A	
	Crenarchaeota (p)	3.6	Anaerobic methanogenic archaeon ET1-8	AJ244284.1	87	Methanogenic cellulose degrader	Chin et al., 1999	N/A	
	Chlorobi (p)	3.0	*I. album* strain JCM 16511	NR_074698.1	83	Facultative anaerobic heterotroph	Iino et al., 2008; Liu et al., 2012	39	99.3
	*Chthonomonadetes* (c)	2.8	Armatimonadetes bacterium JGI 0000077-K19	KJ535399.1	97	Uncharacterized	Nobu et al., 2015	N/A	
	*Acetothermaceae* (f)	2.4	“Ca. A. autotrophicum”	AP011801.1	97	Lithoautotrophic acetogen	Nunoura et al., 2005; Takami et al., 2012	N/A	
	Chlorobi (p)	2.4	*Rhodothermus profundi* strain PRI 2902	NR_116762.1	87	Thermophilic aerobic heterotroph	Marteinsson et al., 2010	N/A	
	*Deltaproteobacteria* (c)	2.4	*Desulfomonile limimaris* DCB-M	NR_025079.1	92	Reductive dechlorinating bacterium	Sun et al., 2001	146	99.3
	*Anaerolineae* (c)	2.4	“*Ca*. Roseilinea gracile” clone JGI24185J35167_10016968	KY937207.1	89	Thermophilic phototroph	Tank et al., 2017	N/A	
	*Anaerolineae* (c)	2.1	*Litorilinea aerophila* strain PRI-4131	NR_132330.1	88	Thermophilic heterotroph	Kale et al., 2013	N/A	
	Chlorobi (p)	1.9	Unidentified bacterium clone K2-30-37	AY344403.2	89	Uncharacterized	Donachie et al., unpublished data	N/A	
	*Syntrophobacteraceae* (f)	1.7	*Thermodesulforhabdus* sp. nov. M40/2 CIV-3.2	AF170420.1	87	Thermophilic sulfate oxidizer	Beeder et al., 1995; Sievert and Kuever, 2000	N/A	
	AC1 (p)	1.3	Unidentified eubacterium from the Amazon	U68651.1	86	Uncharacterized soil bacterium	Borneman and Triplett, 1997	N/A	
		61.1^c^							
Core 2 surface	*Betaproteobacteria* (c)	11.1	*S. paludicola* strain BrT	DQ386858.1	98	Lithoautotrophic Fe(II) oxidizer	Weiss et al., 2007	N/A^f^	
	Chlorobi (p)	9.6	*I. album* strain JCM 16511	NR_074698.1	84	Facultative anaerobic heterotroph	Iino et al., 2008; Liu et al., 2012	N/A	
	*Thaumarchaeota* (c)	6.8	“*Ca*. Nitrosocosmicus sp. Kfb”	KX863712.1	88	Ammonia oxidizer	Alves et al., unpublished data	194	99.6
	*Acetothermales* (o)	5.1	“*Ca*. A. autotrophicum”	AP011801.1	83	Lithoautotrophic acetogen	Nunoura et al., 2005; Takami et al., 2012	N/A	
	*Anaerolineae* (c)	3.4	Chloroflexi bacterium SCGC AAA240-O05	HQ675640.1	88	Uncharacterized	Swan et al., 2011	N/A	
	GAL15 (p)	3.2	*T. ferriorganovorum* strain Z-9801	NR_042719.1	86	Fe(III) reducer	Zavarzina et al., 2002	N/A	
	*Nitrospirales* (o)	3.2	sulfate-reducing bacterium R-PropA1	AJ012591.1	88	Sulfate reducer	Wind et al., unpublished data	N/A	
	Chlorobi (p)	2.1	*I. album* strain JCM 16511	NR_074698.1	83	Facultative anaerobic heterotroph	Iino et al., 2008; Liu et al., 2012	39	99.3
	*Acetothermaceae* (f)	2.1	“*Ca*. A. autotrophicum”	AP011801.1	97	Lithoautotrophic acetogen	Nunoura et al., 2005; Takami et al., 2012	N/A	
	Acidobacteria (p)	2.1	*Thermanaerovibrio* sp. R101	FN556061.1	87	Uncharacterized thermophile	Sayeh et al., unpublished data	N/A	
	*Methanomassiliicoccaceae* (f)	2.1	*Methanomassiliicoccus* sp. N89-2	LN827539.1	92	Methanogen	Huynh et al., 2016	1	99.3
	Chloroflexi (p)	1.9	Chloroflexi bacterium SCGC AAA240-C09	HQ675555.1	88	Uncharacterized	Swan et al., 2011	N/A	
	NC10 (p)	1.7	“*Ca*. Methylomirabilis sp. RS3”	KU891932.1	88	Denitrifying methanotroph	He and Hu, unpublished data	N/A	
	*Anaerolineae* (c)	1.7	*Thermanaerothrix daxensis* strain GNS-1	NR_117865.1	89	Thermophilic anaerobic fermenter	Grégoire et al., 2011	N/A	
	*Chthonomonadetes* (c)	1.5	Armatimonadetes bacterium JGI 0000077-K19	KJ535399.1	97	Uncharacterized	Nobu et al., 2015	N/A	
	*Anaerolineae* (c)	1.5	*Thermomarinilinea lacunifontana* strain SW7	NR_132293.1	91	Thermophilic anaerobic fermenter	Nunoura et al., 2013	N/A	
	*Rhodocyclaceae* (f)	1.3	*Thauera* sp. strain DK5	MF155554.1	95	Denitrifying bacterium	Wang, unpublished data	N/A	
	*Methylomirabiliaceae* (f)	1.3	“*Ca*. M. oxyfera”	FP565575.1	93	Methane oxidizer, nitrate reducer	Ettwig et al., 2010	N/A	
	Acidobacteria (p)	1.3	*Holophaga* sp. WY42	KC921174.1	89	Uncharacterized	Chen, unpublished data	N/A	
	*Thermodesulfovibrionaceae* (f)	1.1	*T. yellowstonii* DSM 11347	CP001147.1	90	Fe(III), sulfate reduction	Henry et al., 1994; Sekiguchi et al., 2008	N/A	
		64.3^c^							
Core 3 surface	*Cenarchaeaceae* (f)	16.5	“*Ca*. Nitrosotenuis sp. AQ6f”	CP024808.1	97	Ammonia oxidizer	Sauder et al., unpublished data	N/A^f^	
	*Syntrophobacteraceae* (f)	10.7	*Thermodesulforhabdus* sp. nov. M40/2 CIV-3.2	AF170420.1	87	Thermophilic sulfate oxidizer	Beeder et al., 1995; Sievert and Kuever, 2000	N/A	
	Chlorobi (p)	9.2	*I. album* strain JCM 16511	NR_074698.1	83	Facultative anaerobic heterotroph	Iino et al., 2008; Liu et al., 2012	39	99.3
	Chlorobi (p)	5.6	*I. album* strain JCM 16511	NR_074698.1	84	Facultative anaerobic heterotroph	Iino et al., 2008; Liu et al., 2012	N/A	
	*Calditrichaceae* (f)	4.1	*Caldithrix abyssi* DSM 13497	CP018099.1	87	Thermophilic anaerobic mixotroph	Miroshnichenko et al., 2003; Kublanov et al., unpublished data	197.1	99.6
	*Betaproteobacteria* (c)	3.8	*S. paludicola* strain BrT	DQ386858.1	98	Lithoautotrophic Fe(II) oxidizer	Weiss et al., 2007	N/A	
	*Nitrospirales* (o)	3.0	Bacterium strain CS35	MG271829.1	87	Uncharacterized	Das and Kerkar, unpublished data	N/A	
	*Pirellulaceae* (f)	2.4	*Thermogutta terrifontis* strain R1	CP018477.1	91	Thermophilic polysaccharolytic bacterium	Elcheninov et al., 2017	35.2	99.6
	Acidobacteria (p)	2.1	*Stenotrophobacter terrae* strain Ac_28_D10	NR_146023.1	96	Aerobic heterotroph	Pascual et al., 2015	N/A	
	*Chitinophagaceae* (f)	1.9	*Cnuella* sp. strain N24	MF797952.1	90	Aerobic heterotroph	Zhao et al., 2014; Wang and Du, unpublished data	N/A	
	Chlorobi (p)	1.7	Unidentified bacterium clone TK-NH7	DQ463733.2	88	Uncharacterized	De Wever et al., unpublished data	N/A	
	*Chthonomonadetes* (c)	1.5	Armatimonadetes bacterium JGI 0000077-K19	KJ535399.1	97	Uncharacterized	Nobu et al., 2015	N/A	
	*Anaerolineae* (c)	1.5	*T. daxensis* strain GNS-1	NR_117865.1	86	Thermophilic anaerobic fermenter	Grégoire et al., 2011	N/A	
	Chloroflexi (p)	1.3	Chloroflexi bacterium SCGC AAA240-C09	HQ675555.1	88	Uncharacterized	Swan et al., 2011	N/A	
	*Pseudanabaenaceae* (f)	1.3	*Pseudanabaena limnetica* PUPCCC 106.2	KM376980.1	95	Thermophilic cyanobacterium	Singh et al., unpublished data	N/A	
	*Nitrospiraceae* (f)	1.3	*Nitrospira* cf. *moscoviensis* SBR2046	AF155155.1	99	Thermophilic nitrite oxidizer	Ehrich et al., 1995; Burrell et al., unpublished data	128.2	98.9
	*Rhabdochlamydiaceae* (f)	1.3	*Rhabdochlamydia crassificans* strain CRIB01	AY928092.1	93	Intracellular pathogen	Corsaro et al., 2007	N/A	
	*Hyphomicrobiaceae* (f)	1.1	*Rhodoplanes* sp. Z2-YC6860	CP007440.1	96	Anoxygenic phototroph	Lee et al., unpublished data	N/A	
	*Phycisphaerae* (c)	1.1	Phycisphaerae bacterium ST-NAGAB-D1	CP019791.1	84	Uncharacterized anaerobe	Spring et al., unpublished data	N/A	
		71.2^c^							

a *Letters in parentheses indicate taxonomic level: k, kingdom; p, phylum; c, class; o, order; f, family; g, genus*.

b *Average percent read abundance across all CP core libraries (n = 42)*.

c *Total percent of reads comprising OTUs with >1% average read abundance*.

d *As determined by NCBI BLASTn*.

e *16S rRNA gene sequences recovered from MAGs using CheckM, and aligned to amplicon sequences using BLASTn*.

f *Not applicable; no 16S rRNA gene sequences from MAGs aligned to this OUT*.

**Figure 2:**
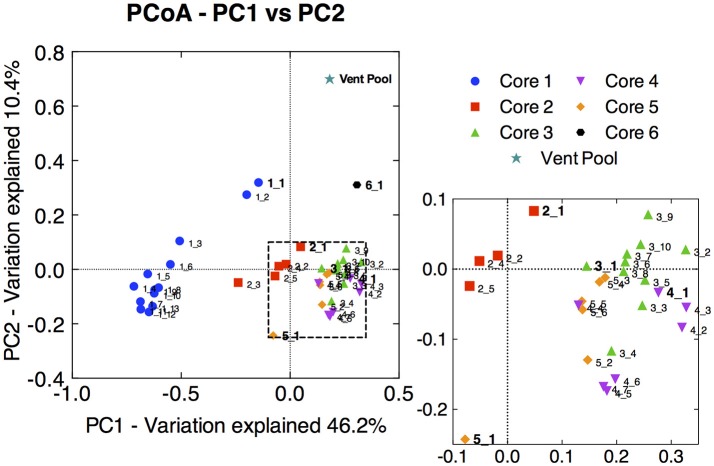
Principal coordinate analysis (PCoA) ordination of pair-wise sample dissimilarity using weighted UniFrac metrics comparing samples from the 16S rRNA gene amplicon library of all CP cores and depth intervals. Zoomed-in panel highlights the less pronounced distribution of samples from cores 3, 4, and 5. Surface sample from each core is labeled in bold, and subsections are labeled with increasing depth. The 16S rRNA gene amplicon library from the CP vent pool water column sample was aligned and normalized to the CP core libraries in order to plot along with the core samples.

Principal coordinate analysis of the 16S rRNA gene libraries revealed a few major trends in microbial community structure within and among the cores. Broadly, microbial communities associated with core 1 were distinct from those associated with core 2, and both were distinct from communities from the distal cores (Figure 2). The variation in community dissimilarity captured by core 1 along the depth transect encompassed that associated with the other cores combined. Within core 1, the samples from the top 2 cm diverged considerably from the deeper samples. Likewise, the surface samples tended to be separate from samples deeper within each core, in particular in cores 2 and 5. This resulted in trajectories in community dissimilarity following a trend with increasing depth (at least as it relates to PC1 and PC2) for cores 1, 2, and 5.

The dominant OTUs across all libraries were related to a crenarchaeote [ca. 9% of total reads, 87% 16S rRNA gene identity (ID) to an anaerobic methanogenic archaeon; Chin et al., 1999), and two Chlorobi (ca. 5% of total reads, each, 84% ID to *Ignavibacterium album;* Iino et al., 2010) (Table 1). The abundant Crenarchaeota OTU was most prevalent in the lower depths of core 1 (below 2 cm) comprising 17.3–41.9% of the reads in the respective libraries; it was also present in the core 2 samples below 1 cm depth (3.4–13.2% read abundance). An additional abundant Crenarchaeota-related OTU was present in only the core 1 samples at all depth intervals at 7.3–12.2% read abundance (Table 1).

The role of Archaea in the CP community remains understudied at this time and requires further analysis in this environment. Several prominent OTUs and MAGs identified as archaeal relatives were identified in the 16S amplicon libraries and metagenomic libraries, respectively. Although the Archaea undoubtedly contributed significantly to the distribution observed in the core samples from the amplicon library, the archaeal OTUs in this study were not related to organisms known to be involved in Fe redox metabolism or CO_2_ fixation. Additionally, putative genes involved in these metabolic systems were not identified in the archaeal MAGs in our study. As such, the Archaea are not a focus for the remainder of this paper.

Although not extremely abundant in the CP community, when all sediment cores were considered together (ca. 0.8% read abundance), OTUs related to *Thermodesulfovibrio* (90% ID to *Thermodesulfovibrio yellowstonii*; Henry et al., 1994; Sekiguchi et al., 2008) are particularly abundant in the topmost layers of core 1 (ca. 6–8% read abundance), less abundant at core 2 (ca. 1–2% read abundance), absent from core 3 (Table 1) and the majority of the core samples from deeper and farther downstream (data not shown). The presence of abundant *Thermodesulfovibrio-*related OTUs in samples from core 1 (CP vent) is not surprising since members of this genus have been shown to reduce Fe(III) (Sekiguchi et al., 2008). Results from previous Fe(III) reducing incubations and recent SIP experiments have suggested that *Thermodesulfovibrio* relatives native to CP may contribute to Fe(III) reduction *in situ* (Fortney et al., 2016, 2018). Additionally, these studies showed decreasing levels of Fe(III) reduction activity with increasing distance from the CP vent. The presence of *Thermodesulfovibrio*-related OTUs in samples from cores 1 and 2, and not in core samples further downstream from CP vent is consistent with results from these studies and together support the potential involvement of *Thermodesulfovibrio* in Fe(III) oxide reduction in CP. Additional abundant OTUs in the core library, including those related to *Acetothermales* (4.7% read abundance)*, Nitrospirales* (3.8% read abundance), and Acidobacteria (3.5% read abundance) tended to be present in greater abundance in the deeper and more distal core samples, and were largely absent from core 1 samples, especially the top few centimeters (Table 1).

Microbial communities in surface samples from cores 2 to 5 exhibited the greatest separation from deeper samples within those respective cores (Figure 2). Notably, these surface communities comprised abundant OTUs (ca. 4–14% read abundance, Table 1) affiliated with the lithoautotrophic Fe(II) oxidizing betaproteobacterium *Sideroxydans paludicola* (98% ID; Weiss et al., 2007). This OTU was largely absent from deeper samples from within the cores suggesting it was a likely driver of the overall separation of the surface and subsurface samples within these cores (Figure 2). The restricted distribution of this OTU in surface samples may be attributable to its dependence on microaerophilic conditions to catalyze Fe(II) dependent growth. The presence of abundant OTUs (4–14% read abundance) related to *Sideroxydans* lends support to the hypothesis that chemolithotrophic Fe(II) oxidation could contribute to Fe redox cycling at CP.

#### CP vent pool

A total of 20,618 high-quality 16S rRNA gene amplicon sequences were obtained from the vent pool sample. Following processing though QIIME a total of 8,587 reads were distributed across 675 OTUs (excluding singletons) at 97% identity. The vent pool was also a diverse microbial community with only 8 OTUs (out of 267 OTUs collapsed to the Family level) with >1% read abundance (Table 2). Together, these OTUs accounted for 43% of all reads. OTUs with unassigned taxa made up 27% of the reads in the vent pool library.

**Table 2:** Microbial community composition of Chocolate Pots vent pool water column.

**SILVA taxonomic assignment^a^**	**% total**	**Representative species match^c^**	**Accession number**	**Similarity (%)**	**Inferred physiology**	**References**	**Representative MAG^d^**	**Similarity (%)**
*Pseudanabaenaceae* (f)	20.1	*Pseudanabaena* sp. 1a-03	FR798944.1	89%	Oxygenic photoautotroph	Cuzman et al., 2010	64%	100%
*Thermodesulfovibrionaceae* (f)	10.3	*Thermodesulfovibrio yellowstonii* DSM 11347	CP001147.1	90%	Fe(III), sulfate reduction	Henry et al., 1994; Sekiguchi et al., 2008	74%	98.8
*Roseiflexaceae* (f)	2.6	*Roseiflexus* sp. RS-1	CP000686.1	99%	Anoxygenic photo(auto)troph	Klatt et al., 2007; van der Meer et al., 2010	N/A^e^	
*Betaproteobacteria* (c)	2.6	*Sideroxydans paludicola* strain BrT	DQ386858.1	99%	Lithoautotrophic Fe(II) oxidizer	Weiss et al., 2007	17%	100%
Chlorobi (p)	2.2	*Rhodothermus profundi* strain PRI 2902	NR_116762.1	87%	Thermophilic aerobic heterotroph	Marteinsson et al., 2010	16%	100%
*Rhodocyclaceae* (f)	2.2	*Thauera* sp. strain DK5	MF155554.1	96%	Denitrifying bacterium	Wang, unpublished data	79%	100%
*Chloroherpetales* (o)	2.1	*Chloroherpeton thalassium* ATCC 35110	NR_074270.1	89%	Anoxygenic photoautotroph	Gibson et al., 1984	54%	100%
*Anaerolineae* (c)	1.3	*Dehalococcoides* sp. BHI80-15	AJ431246.1	88%	Hydrogen oxidizer	Vander Roost et al., 2017	59%	100%
	43.4^b^							

a *Letters in parentheses indicate taxonomic level: k, kingdom; p, phylum; c, class; o, order; f, family; g, genus*.

b *Total percent of reads comprising OTUs with >1% average read abundance*.

c *As determined by NCBI BLASTn*.

d *16S rRNA gene sequences recovered from MAGs using CheckM, and aligned to amplicon sequences using BLASTn*.

e *Not applicable; no 16S rRNA gene sequences from MAGs aligned to this OUT*.

Not surprisingly, the CP vent pool sample was distinct from the core samples (Figure 2). With the exception of relatives of *Sideroxydans* and *Thermodesulfovibrionaceae*, there was no overlap between the abundant OTUs in the CP sediment cores and CP vent pool libraries. A *Pseudanabaenaceae*-related OTU dominated the vent pool community and accounted for 20% of the total reads in the library. The second most abundant OTU was affiliated with *Thermodesulfovibrionaceae* (90% ID to *T. yellowstonii*; Henry et al., 1994; Sekiguchi et al., 2008) at 10% read abundance. The remaining abundant OTUs each comprised about 2% of the total reads in the library and were related to the lithoautotrophic FeOB *Sideroxydans* (99% ID to *S. paludicola*; Weiss et al., 2007) and the anoxygenic phototroph *Roseiflexus* (99% ID to *Roseiflexus* sp. RS-1; Klatt et al., 2007; van der Meer et al., 2010).

*Pseudanabaenaceae* are cyanobacteria that have previously been identified as one of the primary microbial mat-forming species at CP where they form floating streamers at the highest temperature locations (e.g., near the vent, ca. 52°C; Pierson et al., 1999; Pierson and Parenteau, 2000; Parenteau and Cady, 2010). Although less abundant in the amplicon library, *Chloroflexi*, including an OTU related to *Roseiflexus*, (2.6% read abundance, 99% ID to *Roseiflexus* sp. RS-1; Klatt et al., 2007; van der Meer et al., 2010) are also recognized as principal members of the CP mat community (Pierson et al., 1999; Pierson and Parenteau, 2000; Parenteau and Cady, 2010) and thus were also expected in the CP water column. Intriguingly, the presence of relatively abundant OTUs related to *Thermodesulfovibrio* as well as *Sideroxydans* and *Roseiflexus* in the vent pool water column (in addition to the presence of *Thermodesulfovibrio* relatives in the vent sediment) suggests that a coupled Fe redox cycle could be operative at the CP vent.

### Composition of the CP sediment cores and vent pool microbial communities: metagenomic sequence analysis

#### CP sediment cores

The communities inhabiting the top 1 cm depth transects of cores 1, 2, and 3 contained a representative set of OTUs found in all other core libraries based on 16S rRNA gene amplicon analysis. Thus, shotgun metagenomic sequence libraries were obtained from DNA extracted from these three samples. Details on the assembly statistics of the co-assembly can be found in Supplementary Material 1.2. We obtained a total of 167 MAGs from the co-assembly, and the average read coverage of all MAGs in the co-assembly was 15.09. The assemblies were then screened for genes coding for putative EET systems and CO_2_ fixation pathways to assess the possibility of Fe redox cycling and contribution to primary production in CP.

**Figure 3:**
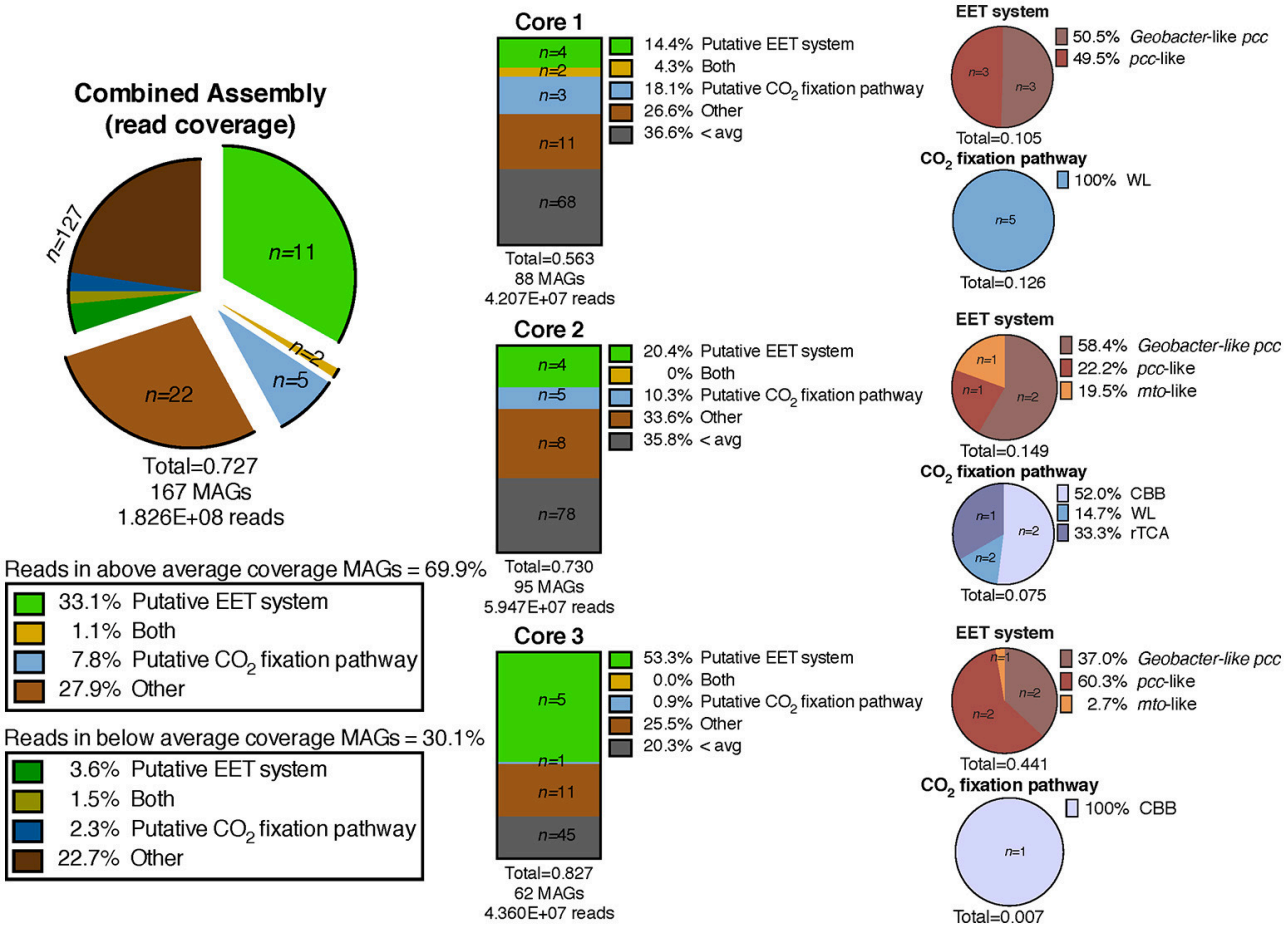
Exploded pie chart on the left shows a distribution of MAGs and percentage of metagenomic sequence reads mapped to MAGs containing metabolic pathways of interest. Middle bars represent the MAGs identified in the three individual core metagenomes and the numbers of MAGs containing pathways of interest and percentage of reads mapped to those MAGs. Pie charts on the right show the breakdown of specific EET systems or CO_2_ fixation pathways present in each core sample. The total listed below each pie and bar chart represents the ratio of mapped reads in a given metagenomic assembly or pathway to the total number of mapped reads for that assembly. EET, extracellular electron transfer; CBB, Calvin-Benson-Bassham cycle; WL, Wood-Ljungdahl pathway; rTCA, reductive tricarboxylic acid cycle; 3HP, 3-hydroxypropionate bicycle.

We defined “abundant” MAGs as those with higher than average read coverage, and focused on these for further analysis. From the co-assembly of the three metagenomes, 11 abundant MAGs were shown to encode putative EET systems (see section Materials and Methods), four encoded putative CO_2_ fixation pathways, and three MAGs encoded both. These 18 MAGs accounted for >40% of total mapped reads, suggesting the high abundance of populations represented by these MAGs, and the importance of putative EET and CO_2_ fixation processes in the environment. The remaining abundant MAGs (*n* = 22, 27.9% mapped reads, Figure 3) did not contain either pathway and these organisms were not considered further in this study. MAGs with below average coverage (*n* = 127, 30.1% mapped reads) were also not subjected to further analysis. The microbial community of each core was considered individually in addition to the co-assembly in order to determine how the metabolic potential changes with distance moving away from the hot spring vent (Figure 3). Core 1 contained 88 MAGs with an average normalized coverage of 7.94, core 2 contained 95 MAGs with an average normalized coverage of 9.75, and core 3 contained only 62 MAGs with an average normalized coverage of 11.27.

**Figure 4:**
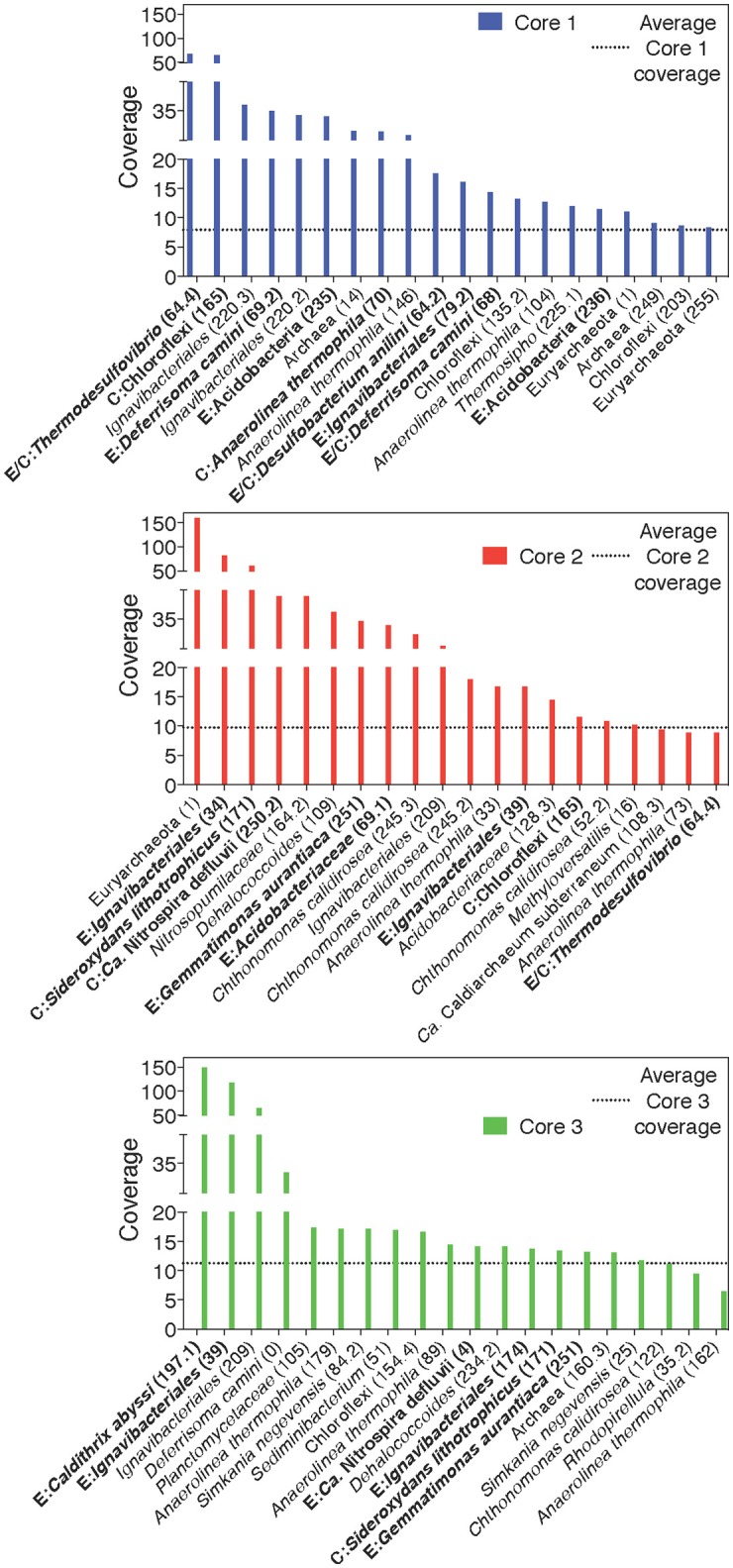
Rank-abundance plots of cores 1, 2, and 3 highlight the 20 most abundant taxa within each core sample. An average normalized read coverage of 7.94, 9.75, and 11.27 for MAGs in cores 1, 2, and 3, respectively, is marked with a horizontal dotted line. MAGs containing putative EET systems or CO_2_ fixation pathways are bolded and labeled with and E or C, respectively, or both for MAGs containing both putative metabolisms. MAG numbers are listed in parentheses.

**Figure 5:**
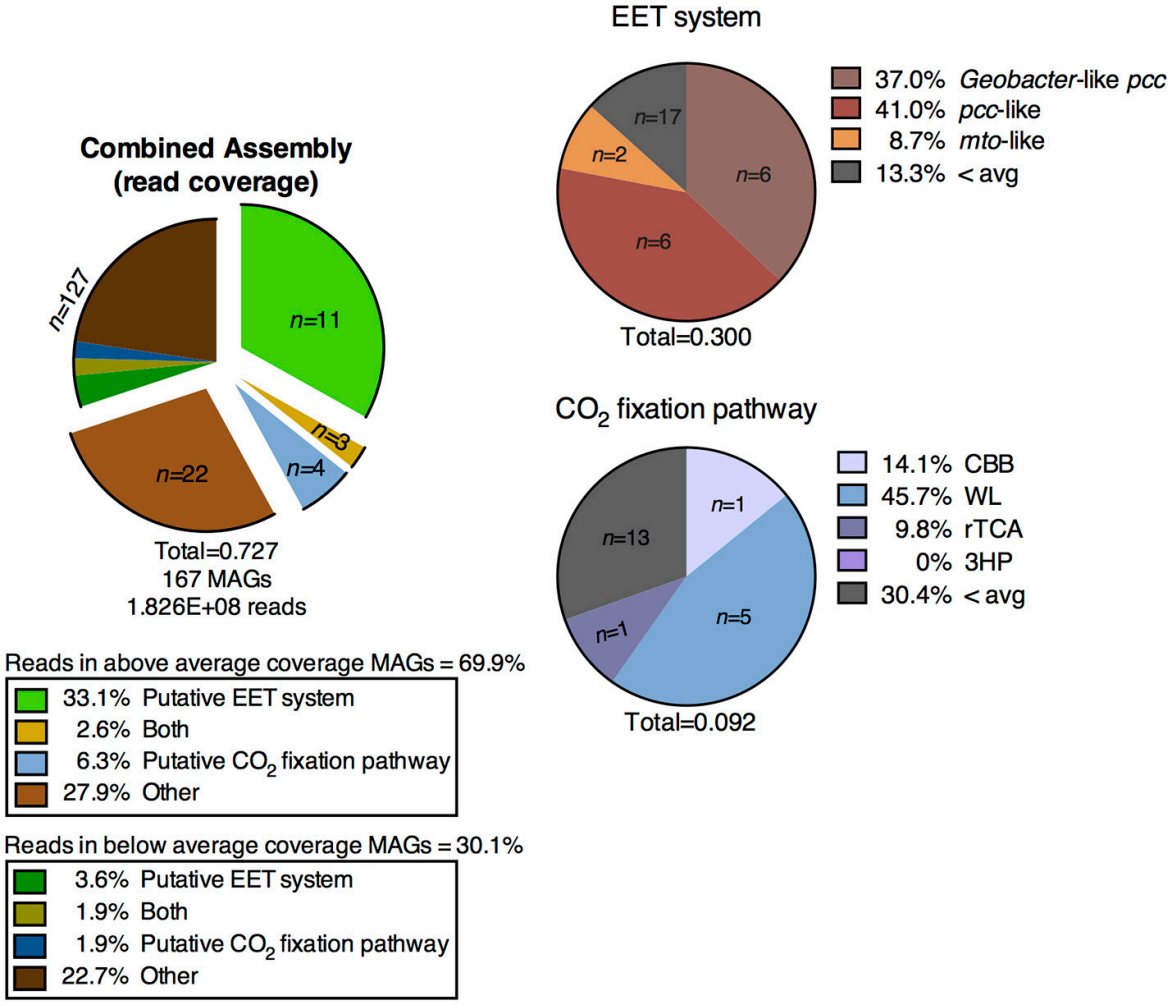
Distribution of MAGs from the metagenomic co-assembly of the CP cores containing putative metabolic pathways of interest, and percentage of metagenomic reads mapped to those MAGs. EET, extracellular electron transfer; CBB, Calvin-Benson-Bassham cycle; WL, Wood-Ljungdahl pathway; rTCA, reductive tricarboxylic acid cycle; 3HP, 3-hydroxypropionate bicycle.

A separation of microbial communities from the three cores was evident both when considering the collection of MAGs as a whole and in relation to MAGs containing either a putative EET system or CO_2_ fixation pathway (Figures 3–5). The high-coverage MAGs from the metagenomic libraries were representative of the abundant OTUs from the amplicon libraries (Supplementary Table 2). Core 1 was predominantly composed of Chloroflexi, *Ignavibacteriales, Thermodesulfovibrio*, Acidobacteria, and *Deferrisoma*. Chloroflexi are known members of the microbial mat community at CP and not unexpected in the core 1 sediment (Parenteau and Cady, 2010). *Thermodesulfovibrio*, Acidobacteria, and *Ignavibacteriales* have all been previously cited as principal members of the CP Community (Fortney et al., 2016, 2018; Figure 4, Supplementary Figures 3, 4). Aside from the archaeal MAGs, core 2 also contained high-coverage *Ignavibacteriales* and *Sideroxydans* MAGs. Core 3 comprised a high-coverage *Caldithrix* MAG, two MAGs related to *Ignavibacteriales* and a *Deferrisoma* MAG. A number of high-coverage MAGs related to *Ignavibacteriales*, Acidobacteria, *Caldithrix*, and *Deferrisoma* encoded putative EET systems and were distributed between different core samples. This observation, when coupled with the documented Fe(III) reduction activity at core sites 1 and 3 (see Fortney et al., 2018), suggests that the Fe(III) reducing community at CP is complex and diverse.

Cores 1 and 2 presented more similarity in terms of MAGs encoding putative EET systems than either individual community had with that of core 3 (Figure 4, Supplementary Figure 3). While the overall number of MAGs encoding a particular EET system was similar between the cores (Figure 3), in core 3 over 50% of all assembled reads mapped to MAGs containing putative EET systems. In contrast, for cores 1 and 2, only 20% of the assembled reads were mapped to EET-containing MAGs. This is an interesting result considering the activity levels of Fe(III)-reduction observed in previous studies (Fortney et al., 2018). For example, Fe(III)-reduction is more active at the CP vent (i.e., core 1) whereas the genomic potential for Fe(III)-reduction (e.g., the presence of a putative EET system) is more evident at core 3. Not only was the overall read abundance of EET-containing MAGs driving the separation between cores 1, 2, and 3 but the distribution of abundant MAGs, most of which contained putative EET systems, is also a likely driver.

#### CP vent pool water column

A shotgun metagenomic sequence library was obtained from DNA extracted from membrane filters collected from the vent pool at CP. The average adjusted coverage (see Supplementary Text 1.2) of all MAGs in the metagenomic assembly was 28.61. The high-coverage MAGs from the metagenomic library were representative of the abundant OTUs from the 16S rRNA gene amplicon library (Supplementary Table 3). Eleven MAGs had an above-average coverage and comprised 79.2% of the mapped metagenomic reads. One MAG encoded a putative EET system and two MAGs encoded a putative CO_2_ fixation pathway; these two MAGs comprised over 20% of the mapped reads in the entire metagenomic assembly. One MAG encoded both systems. The remaining high-coverage MAGs did not encode metabolic pathways directly relevant to Fe cycling and thus were not considered further in this study. The below-average coverage MAGs (*n* = 32, 20.2% mapped reads) which did not encode putative EET systems or CO_2_ fixation pathways are also not considered in the remainder of this study.

**Figure 6:**
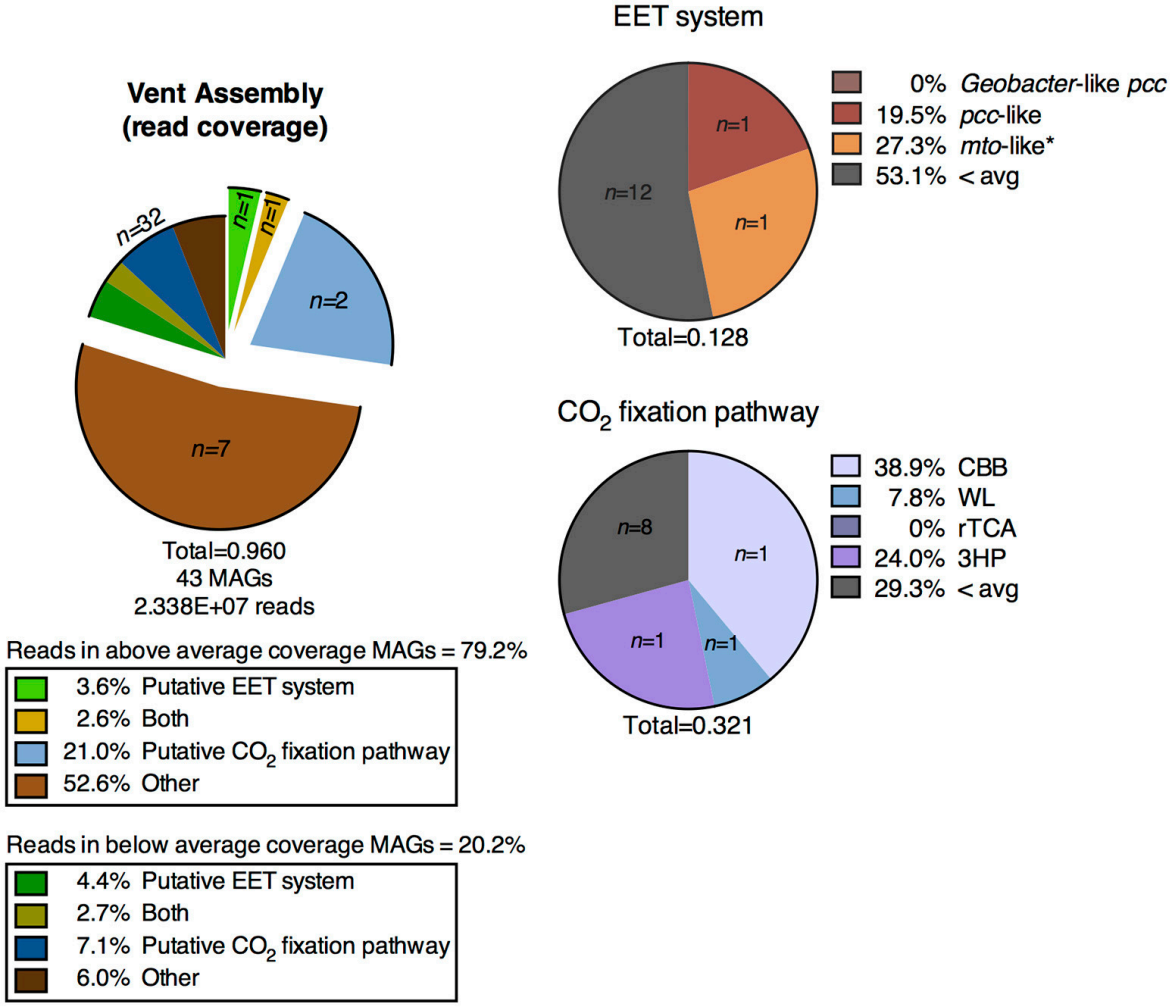
Distribution of MAGs from the metagenomic assembly of the CP vent pool water column containing putative metabolic pathways of interest and the percentage of metagenomic reads mapped to those MAGs. EET, extracellular electron transfer; CBB, Calvin-Benson-Bassham cycle; WL, Wood-Ljungdahl pathway; rTCA, reductive tricarboxylic acid cycle; 3HP, 3-hydroxypropionate bicycle.

EET systems are much less prevalent in the CP vent pool water column metagenomic assembly than in the surface samples from sediment cores 1, 2, and 3. Less than 4% of the metagenomic reads in the vent pool water column assembly mapped to MAGs containing these pathways (Figure 6), as compared to >30% in the CP core co-assembly (Figures 3, 5). The type of putative EET system was also quite different between the metagenomic assemblies; in particular there was a lack of high-coverage MAGs encoding a *Geobacter*-like *pcc* system in the CP vent pool (Figure 6).

Carbon dioxide fixation was a prominent metabolic process in the vent pool water column metagenome. Approximately 24% of the metagenomic reads mapped to only three MAGs containing these putative metabolic systems (Figure 6). The highest read-coverage MAG was related to the cyanobacterium *Pseudanabaena* and encoded a full CBB cycle. No MAGs encoded alternative archaeal pathways utilizing RuBisCO nor did any high-coverage MAGs encode a complete putative rTCA cycle. Although, two Chlorobi related MAGs encoded a partially complete rTCA cycle, which is expected for members of this phylum. A partially complete 3HP bicycle was identified in one MAG related to *Roseiflexus* (Supplementary Figure 4). Putative CO_2_ fixation pathways were identified in additional lower-coverage MAGs including a relative of *Sideroxydans*.

The highest coverage MAG after the *Pseudanabaena* relative was a Chlorobi identified as *Pelodictyon*. An additional Chlorobi, *Chloroherpeton*, was present in the vent pool water column metagenomic assembly, though at a more moderate read-coverage (Supplementary Figure 3). While *Pelodictyon* (now called *Chlorobium*; Imhoff, 2003) and *Chloroherpeton* have not specifically been identified at CP before, anoxygenic phototrophic Chlorobi related to “*Candidatus* Thermochlorobacter aerophilum” have been previously described as part of the microbial mat community (Klatt et al., 2013). A high-coverage *Pseudanabaena* MAG was unsurprising given its abundance as part of the mat community at CP (Parenteau and Cady, 2010, and references therein). As described above in reference to the OTU libraries, multiple MAGs related to Chloroflexi, *Thermodesulfovibrio*, and *Ignavibacteriales* were identified in the CP vent pool metagenomic library and are expected members of the microbial community. Although multiple MAGs of the aforementioned taxa were identified in the metagenome, only one particularly high-coverage representative MAG of each organism was present (Supplementary Figure 4, Supplementary Table 3).

One of the guiding hypotheses of this study was that putative lithoautotrophic FeOB are present and active at CP. Given that the *Sideroxydans* MAG in the CP vent pool metagenomic assembly had only slightly below-average read coverage, it would appear that this group of organisms may have a modest presence in CP vent pool microbial community. Metagenomic read coverage and inferred microbial abundance based on 16S rRNA gene amplicon OTU abundance track reasonable well (Tables 1, 2, and Supplementary Tables 2, 3). In previous Fe(III)-reducing incubation experiments a high abundance of putative FeRB correlated with high levels of Fe(III) reduction activity(Fortney et al., 2016, 2018). However, it is necessary to point out that in terms of the energetics of Fe-based microbial metabolisms, Fe(III) reduction yields greater free energy for cell processes, including cell division, than does Fe(II) oxidation (Neubauer et al., 2002; Bird et al., 2011). Even if FeRB and FeOB have equivalent levels of activity, e.g., the same number of moles of Fe metabolized, one would expect a lower cell density of FeOB simply because of the lower energy potential of the metabolic reaction. Nevertheless, as is detailed below, the metabolic potential of this MAG along with its inferred phylogeny supports the hypothesized presence of lithoautotrophic FeOB at CP.

### Presence of putative EET systems at CP and the potential for Fe(III) reduction

It is important to acknowledge that although several MAGs in both metagenomic assemblies contain putative EET systems, the presence of these gene homologs does not in and of itself prove the existence of Fe(III) reduction activity (Shi et al., 2014). However, the previously documented Fe(III) reduction activity from materials collected from these locations at CP (Fortney et al., 2018), coupled with the genomic results identifying the metabolic potential for EET systems, support the hypothesis that these taxa are involved in Fe(III) reduction *in situ*. In contrast, experimental evidence for lithoautotrophic Fe(II) oxidation is currently not available and the operation of this metabolic pathway at CP is more speculative.

#### CP cores

Sequences homologous to the porin from the well characterized *Geobacter*-like *pcc* EET system were identified in several abundant MAGs in the metagenomic co-assembly, whereas homologs to the *Shewanella*-like *mtrABC* or any of the model Fe(II)-oxidizing EET systems were not identified in the metagenomic co-assembly of the cores. Searches for non-model EET systems identified *pcc*-like systems in abundant MAGs from all three cores and *mto*-like systems in abundant MAGs from all cores (Figures 3, 5, Supplementary Figure 3).

Homologs to the *Geobacter*-like *pcc* porin (Liu et al., 2014; Shi et al., 2014) were located in MAGs identified *Ignavibacteriales* (*n* = 7) and *Deferrisoma camini* (*n* = 2; Supplementary Figure 3). The genome of *Ignavibacterium album* is known to encode a *Geobacter*-like *pcc*-porin (Shi et al., 2014); the same is true for the related *Ignavibacteriales* species, *Melioribacter roseus* (Fortney et al., 2016). The three *Ignavibacteriales* MAGs identified as having only a partially complete *Geobacter*-like *pcc* system are missing a homolog to *gsu1999*, an additional periplasmic *c-*cyt predicted to be in this EET system (Santos et al., 2015; Shi et al., 2016). It should be noted that while the *Ignavibacterium* genome is expected to encode this homolog, the *Melioribacter* genome is not known to encode this gene as part of its *Geobacter-*like *pcc* system, and *Melioribacter* is still capable of carrying out Fe(III) reduction (Podosokorskaya et al., 2013). *Deferrisoma* spp. are known to be FeRB (Slobodkina et al., 2012; Pérez-Rodríguez et al., 2016), and although the exact mechanism for Fe(III) reduction has not been described in this organism, the published genome for *D. camini* S3R1 encodes a homolog of the *Geobacter-*like *pcc*-porin (IMG gene ID 2517273319) and accompanying *c*-cyts that were predicted in the EET system model (Shi et al., 2016). Although the *c-*cyts in *D. camini* were predicted to be only periplasmic, extracellular *c-*cyts were detected elsewhere in the genomes. Unexpectedly, we also identified the metabolic potential for autotrophic Fe(III) reduction in one *D. camini* MAG, that is, the presence of both a putative EET system and CO_2_ fixation pathway (see “Presence of putative CO_2_ fixation systems” section below, and Supplementary Figure 3). While not observed in *D. camini*, a related *Deferrisoma* sp. has previously demonstrated this capability (Pérez-Rodríguez et al., 2016).

Putative EET systems that were not homologous to the *Geobacter-*like *pcc-*system were detected in MAGs identified as “*Candidatus* Nitrospira defluvii,” Acidobacteria (*n* = 3), and *Desulfobacterium anilini*. A *pcc-*like EET system was also detected in the *Caldithrix* MAG, and while *Caldithrix* spp. are not known to be FeRB (Miroshnichenko et al., 2003, 2010; Kublanov et al., 2017), the ability to use Fe(III) as a terminal electron acceptor has not been explicitly tested in these organisms. In any case, the published genome for *Caldithrix abyssi* LF13 also encodes a homolog of the *Geobacter-*like *pcc* porin (IMG gene IDs 2720325731) as well as the predicted associated *c-*cyts. “*Ca*. N. defluvii” and *Desulfobacterium* spp. are known as nitrite oxidizing bacteria and sulfate reducing bacteria, respectively (Brysch et al., 1987; Lücker et al., 2010; Suzuki et al., 2014). *D. autotrophicum* is capable of reducing Fe(III), although not as a means of respiration (Lovley, 2006), and a similar process may be taking place here. The detection of putative EET systems in the Acidobacteria MAGs is consistent with previous data indicating that organisms within this lineage (e.g., *Geothrix fermentans* and *Thermoanaerobaculum aquaticum*) can reduce Fe(III) (Coates et al., 1999; Losey et al., 2013). Acidobacteria have also been identified in metagenomic assemblies of Fe(III) enrichment cultures (Fortney et al., 2016) and Fe(III) reducing incubations derived from CP (Fortney et al., 2018).

The MAG identified as *Gemmatimonas aurantiaca* encoded an *mto-*like EET system, although it is necessary to reiterate that our classification of “*mto*-like” simply refers to the lack of an identified extracellular *c-*cyt that is predicted for Fe(III) reducing EET systems as opposed to any specific knowledge about the metabolic potential of a particular MAG. The representative isolate *G. aurantiaca* T-27^T^ has not been specifically investigated for its ability to oxidize Fe(II) or reduce Fe(III) (Zhang et al., 2003). Curiously, the two *Thermodesulfovibrio* MAGs (although only one MAG was particularly abundant) also encoded putative “*mto-*like” EET systems. However, a possible explanation for the “missing” extracellular *c-*cyt, as is also potentially the case for the *Gemmatimonas* MAG, could be due to the metagenomic assembly and binning process, which failed to generate contigs containing this gene. This is especially likely given the previous identification of the *Geobacter*-like *pcc* and *pcc*-like EET systems in MAGs related to *Thermodesulfovibrio* (Fortney et al., 2016, 2018) and, as is discussed below, the identification of a complete *pcc-*like EET system in the CP vent pool *Thermodesulfovibrio*-like MAG (Supplementary Figure 4).

It was surprising to determine that the *Sideroxydans* MAG did not contain any evidence for an EET system. No 16S rRNA gene was recovered from this MAG so it cannot be specifically related back to the 16S rRNA gene amplicon library of the sediment core samples. However, based on a similar change in relative abundance/coverage between the sediment cores and the phylogenetic identity of this MAG, we can reasonable conclude this MAG derives from the same organism. Given the close relatedness of the 16S rRNA gene amplicon to the known FeOB *S. paludicola*, we would expect the MAG to present the same putative metabolic potential. The lack of detection of an EET system in this MAG suggests that it may be differentiated from *S. paludicola* metabolically.

#### CP vent pool water column

Two above-average coverage MAGs from the vent pool metagenome encoded putative EET systems. An *mto*-like EET system was identified in a MAG belonging to the *Ignavibacteriales*, and a *pcc*-like EET system was identified in a *Thermodesulfovibrio* relative. The presence of putative EET systems in MAGs related to either of these taxa is consistent with our previous work (Fortney et al., 2016, 2018). However, the putative EET system in the *Ignavibacteriales* MAG is identified as “*mto-*like.” Although as discussed above, this classification refers to the lack of an extracellular *c*-cyt proximal to the putative porin and may simply represent an incomplete EET system.

It is interesting to note that several putative EET systems, both *Geobacter*-like *pcc* and *pcc*-like, were identified in low coverage MAGs including multiple *Thermodesulfovibrio, Ignavibacteriales*, and *Deferrisoma* MAGs, among others (Supplementary Figure 4). In contrast to the MAG from the CP sediment core metagenomic co-assembly, the *Sideroxydans* MAG from the CP vent pool metagenomic assembly encoded a putative EET system. However, it was identified as *pcc-*like and shared no homology to the *mtoABCD* system, which is expected for *Sideroxydans* spp. (Emerson et al., 2013). Metabolic differentiation between planktonic and sediment microbial communities at CP is potentially based on the geochemical differences between the solid phase (i.e., sediment) and dissolved species in the aqueous phase. This is consistent with the observations made at other hot springs in Yellowstone (Colman et al., 2016, and references therein). Qualitatively, the CP vent pool has been observed to be dynamic and well mixed. However, high-resolution measurements of geochemical gradients within the water column may provide additional insight into the differentiation between planktonic and sediment microbial communities.

### Presence of putative CO_2_ fixation systems at CP and the potential for litho- or photo-autotrophy

#### CP cores

Overall, MAGs encoding putative CO_2_ fixation pathways were less abundant than those encoding putative EET systems (Supplementary Figure 3). CO_2_ fixation appeared to be a less prevalent metabolic process in the CP sediment core system, especially in core 3, as compared to potential Fe-based metabolisms (i.e., MAGs containing an EET system) (Figure 5). Genes encoding the WL pathway were the most abundant in terms of both the number of MAGs encoding a complete CO_2_ fixation pathway and the high percentage of metagenomic reads which mapped to these MAGs relative to other putative CO_2_ fixation pathways. Genes encoding the WL pathway were detected in MAGs identified in core 1 (*n* = 5) and core 2 (*n* = 1) while genes encoding the CBB pathway were detected in one MAG identified in both sediment cores 2 and 3. Genes encoding for the rTCA pathway were detected in a single MAG in core 2 (Figure 3). One archaeal MAG encoded a homolog to thiazole-adenylate synthase, the alternate ribulose bisphosphate regenerating enzyme proposed by Finn and Tabita (2004); however this MAG was only partially complete as sedoheptulose-1,7-bisphosphatase, a key enzyme in the pathway, was not detected. We were unable to identify a complete 3HP bicycle in any of the MAGs from the metagenomic co-assembly. Only one moderately abundant MAG identified as a relative of *Dehalococcoides* from core 3 coded for a partial 3HP pathway (Supplementary Figure 3). However, this MAG did not code for a homolog of malonyl-CoA reductase (EC:1.2.1.17), a key marker gene predicted to be in the pathway. This apparent absence of a complete 3HP pathway in this MAG is consistent with the previous suggestion that members of this genus do not encode this pathway (Hügler and Sievert, 2011).

As expected based on the genome sequence of *Sideroxydans lithotrophicus* ES-1 available on IMG (genome ID 646564569) and previous studies of *Sideroxydans* spp. (Weiss et al., 2007; Emerson et al., 2013), the *Sideroxydans* MAG identified in the CP sediment cores encoded a full CBB cycle. However, the detection of a complete CBB pathway in one of the low-coverage *Thermodesulfovibrio* MAGs was unexpected since *Thermodesulfovibrio* spp. are not known to be autotrophic (Henry et al., 1994; Sekiguchi et al., 2008; Orcutt et al., 2015).

Genes encoding a full WL pathway were identified in five MAGs (Figure 5). Although one MAG was identified as *D. anilini*, it has since been reclassified as the genus *Desulfatiglans* (Suzuki et al., 2014). Its distant relative, *Desulfobacterium autotrophicum*, has been shown to use the WL pathway to fix CO_2_ (Schauder et al., 1989). While the two genera are distinct (ca. 85% 16S rRNA gene sequence similarity) and *Desulfatiglans* are not known to be autotrophic (Suzuki et al., 2014). *Desulfatiglans* and *Desulfobacterium* are both members of the family *Desulfobacteraceae*, and an operative WL pathway has been identified in several other species within this family, including Desulfonema, Desulfosarcina (Kuever et al., 2005), *Desulfospira* (Finster et al., 1997), and *Desulfotignum* (Kuever et al., 2001; Schink et al., 2002; Ommedal and Torsvik, 2007). The evolutionary history of these organisms may offer an explanation for why a putative WL pathway was also detected in the *Desulfatiglans* MAG. Furthermore, heterotrophic acetate assimilation has been shown to occur using the WL pathway run in reverse (oxidative acetyl-CoA pathway) (Schauder et al., 1989; Hattori et al., 2005; Can et al., 2014); this is also the case for several of the aforementioned *Desulfobacteraceae* (Kuever et al., 2005). This is a possible explanation for the detection of genes encoding the WL pathway in MAGs identified as Chloroflexi and *Deltaproteobacteria*, both of which are known to encode the WL pathway (Hügler and Sievert, 2011; Can et al., 2014). Heterotrophic metabolism via the oxidative acetyl-CoA pathway additionally offers an explanation for the detection of genes encoding a full WL pathway in one of the MAGs identified as *Thermodesulfovibrio*. An incomplete WL pathway [lacking carbon monoxide dehydrogenase (CODH)] has been detected in other *Thermodesulfovibrio* spp. (Henry et al., 1994; Frank et al., 2016) and it is plausible that the *Thermodesulfovibrio*-relatives native to CP have acquired the missing CODH gene through horizontal gene transfer. Further investigation is required to fully resolve the metabolic capabilities of these organisms.

Genes encoding ATP-citrate lyase (*aclAB*) have been used previously as genetic markers of the rTCA cycle in microbial communities (Hügler et al., 2005). However, caution has been stressed in using *aclAB* alone as indication for the presence of rTCA (Williams et al., 2006). More recent studies have identified additional mechanisms that bacteria can use to cleave citrate (i.e., citryl-CoA synthase and citryl-CoA lyase, see Supplementary Text 1.1 for details) along with other enzymes (i.e., 2-oxoglutarate synthase) that can catalyze the irreversible reactions unique to the pathway (Hügler and Sievert, 2011). For these reasons, we took a conservative approach when looking for the presence of the key marker genes along with all other genes predicted in the pathway as a positive indication for the rTCA cycle in a bin. As a result the high-coverage “Ca. Nitrospira defluvii” MAG was the only positive identification of a full rTCA cycle, which is consistent with previous reports of this pathway in “Ca. N. defluvii” (Lücker et al., 2010).

Definitive abundant phototrophic MAGs were not present in the CP sediment core metagenomic co-assembly. Genes encoding PS-II and -I were detected in cyanobacterial MAGs (e.g., *Oscillatoriales, Pseudanabaena*, and *Synechococcus*). However, these MAGs had very low read-coverage (ca. 2–5x) and were not considered further in this study.

#### CP vent pool water column

The two cyanobacterial MAGs, *Synechococcus* and *Pseudanabaena* coded for full a CBB cycle and complete PS-II and -I gene complex. The *Sideroxydans* MAG also encoded a full CBB pathway (Supplementary Figure 4), as expected based of previous genomic characterization of this genus (see above). A single abundant MAG, *Thermodesulfovibrio*, encoded a full WL pathway. As is described above in regard to the CP sediment core metagenomic co-assembly, this *Thermodesulfovibrio-*relative may have acquired CODH through horizontal gene transfer, although further phylogenetic analysis is needed to evaluate this possibility. Even though the *Pelodictyon* and *Chloroherpeton* MAGs only encoded partial rTCA cycles, they also encoded homologs of anoxygenic photoreaction centers (Supplementary Figure 4); anoxygenic photoautotrophy via rTCA is expected for members of the *Chlorobiaceae* (Hügler and Sievert, 2011). The 3HP cycle was proposed for and characterized in *C. aurantiacus* (Strauss and Fuchs, 1993; Zarzycki et al., 2009). Genes involved in this autotrophic pathway have since been identified in related Chloroflexi, i.e., *Roseiflexus* spp. (van der Meer et al., 2010), and stable isotope probing experiments have indicated the potential for CO_2_ fixation via 3HP (Klatt et al., 2007). The putative 3HP bicycle in the *Roseiflexus* MAG is only partially complete, however given the aforementioned information; it is not unexpected for this organism.

### Evidence for a coupled Fe redox cycling microbial community at CP

Although this study took a bioinformatics approach to probing the *in situ* microbial community for evidence for Fe redox cycling, it is important to recall that previous enrichment culturing (Fortney et al., 2016) and incubation studies (Fortney et al., 2018) have experimentally demonstrated the Fe(III) reducing capability of the CP microbial community. These observations, combined with the genomic evidence for the metabolic potential for EET and Fe(III) reduction as presented here (Figure 5, Supplementary Figure 3), allows us to confidently assert that Fe redox cycling is an important process supporting microbial metabolism in CP.

As for the oxidative side of the Fe cycle, putative FeOB (i.e., *Sideroxydans* MAGs) were detected in both the sediment and planktonic components of the CP microbial community, and genomic evidence indicates their potential contribution to lithoautotrophic Fe(II) oxidation (Supplementary Figures 3, 4). The relatively low metagenomic coverage of these MAGs (at least in the CP vent pool water column) is reasonable given the expected lower energy yields of this Fe(II) oxidation (Bird et al., 2011). The *in situ* activity of putative FeOB warrants further direct investigation (e.g., transcriptomics), and despite the relatively low abundance these MAGs, it is possible that they have a nontrivial contribution to Fe(II) oxidation and CO_2_ fixation *in situ. Sideroxydans* spp. are microaerophiles (Neubauer et al., 2002; Emerson and Weiss, 2004) and the low O_2_ concentrations measured at the CP vent, ca. 0–5% air saturation (Roden, unpublished data; Wu et al., 2013), are amenable to growth of these organisms. Dissolved oxygen never reaches supersaturation in the spring water in the CP flow path nor within the microbial mats (Pierson et al., 1999; Parenteau et al., 2014), however higher concentrations of O_2_ have been measured in the vent pool, ca. 25% air saturation (Pierson et al., 1999), which could be toxic to these cells and may have an impact on their overall abundance. This information, combined with that from previous studies of the potential for lithoautotrophic Fe(II) oxidation activity at CP (Trouwborst et al., 2007) as well as unsuccessful attempts at culturing these organisms (Emerson and Weiss, 2004), suggests that the majority of Fe(II) oxidation at CP is due to abiotic oxidation by biogenic O_2_ produced by Cyanobacteria. We thus conclude that the vast majority of Fe(II) oxidation occurs as an indirect result of the production of O_2_ by Cyanobacteria in the community, a conclusion that is consistent with those made previously (Pierson et al., 1999; Pierson and Parenteau, 2000; Emerson and Weiss, 2004; Trouwborst et al., 2007; Parenteau and Cady, 2010).

In addition to indirect Fe(II) oxidation, Cyanobacteria undoubtedly have the greatest impact on fixed carbon within the water column, mat, and sediment environments at CP. There is still some uncertainty as to the ability of *Roseiflexus* to fix CO_2_ (Klatt et al., 2007; van der Meer et al., 2010; Tang et al., 2011; Tank et al., 2017), and while the abundant MAGs of other anoxygenic phototrophs, *Pelodictyon* and *Chloroherpeton*, encoded only partial putative CO_2_ fixation pathways, the rTCA cycle is known to be operative in the Chlorobi (Frigaard and Bryant, 2008). Members of these photoautotrophic phyla have previously been identified at CP (Klatt et al., 2013; Fortney et al., 2018) and are all likely contributing substantially to the fixed carbon budget that is in turn supplying the heterotrophic FeRB community at CP.

It is entirely possible that *Sideroxydans*, as well as the rest of the CP community fluctuates temporally or spatially, however without more data we can merely speculate at this time. A protracted sampling campaign to assess diurnal and even seasonal cycles could illuminate whether the abundant organisms found in this study consistently dominate the microbial community, or if they are subject to significant temporal variations. Due to the unsuccessful attempts to study the FeOB community at CP using culturing (Emerson and Weiss, 2004) or stable isotope probing techniques (Fortney et al., unpublished results), future investigations will almost certainly require culture-independent techniques (e.g., transcriptomics) to measure levels of abundance and activity of the Fe cycling microbial community at CP.

### Comparison of CP to other circumneutral Fe-rich seep/spring environments

In many ways CP resembles other circumneutral-pH Fe seep (Haaijer et al., 2008; Blöthe and Roden, 2009; Roden et al., 2012) and Fe-rich spring-like environments (Hegler et al., 2012; Ward et al., 2017), where Fe(II)-rich subsurface fluids contact atmospheric oxygen, resulting in the accumulation of Fe(III) oxide deposits. The results of our incubation studies and metagenomic investigations are consistent with other studies that have demonstrated the potential for such oxide deposits to serve as electron acceptors for FeRB (Emerson and Revsbech, 1994; Haaijer et al., 2008; Blöthe and Roden, 2009; Hegler et al., 2012; Roden et al., 2012). However, a notable characteristic that sets CP apart from these other ecosystems is the absence of abundant putative FeOB in the spring water near the vent source. One might attribute this difference to the mildly thermophilic conditions at the CP vent (ca. 50°C), which is significantly warmer than canonical neutrophilic FeOB (e.g., *Sideroxydans*) habitats (Emerson et al., 2013). However, *Sideroxydans*-related sequences have been identified in a Japanese thermal spring (ca. 45°C) similar to CP (Ward et al., 2017). The extent to which these ecosystems are exposed to direct sunlight, and therefore the presence or absence of phototrophs (e.g., Cyanobacteria), may have a pronounced effect on the Fe-oxidizing microbial community. Cyanobacteria are absent from the Jackson Creek Fe seep environment in Indiana where tree canopy cover prevents abundant growth of phototrophic microorganisms, and the main O_2_ input is from the atmosphere (Roden et al., 2012). In contrast, CP is fully exposed and hosts an abundant phototrophic community comprised of Cyanobacteria, Chlorobi and Chloroflexi in both microbial mats and planktonic phases (Parenteau and Cady, 2010; Supplementary Figure 4). In this way CP is analogous to other Fe-rich spring systems in that the Cyanobacteria mat communities are spatially segregated to the margins of the vent pool (Hegler et al., 2012) and flow path further downstream (Ward et al., 2017).

Ultimately, it is a combination of factors (e.g., flow rate, insolation, temperature, oxygen saturation) in these circumneutral-pH Fe-rich ecosystems that control microbial community composition, and therefore the Fe redox cycling metabolic pathways that are present and active in each of these environments. The reason for the diminished role of FeOB in the CP vent pool water column is not clear at this time. Further analysis of this hot spring and other Fe-rich seep/spring-like environments is needed to resolve these differences.

## Author contributions

NF, ER, and EB designed the research. NF, ER, EB, and BC conducted fieldwork. Laboratory work was conducted by NF and BC. Data was analyzed by NF and SH. NF wrote the manuscript with help and input from ER, EB, SH, and BC.

### Conflict of interest statement

The authors declare that the research was conducted in the absence of any commercial or financial relationships that could be construed as a potential conflict of interest. The handling editor declared a past co-authorship with one of the authors ER.
