# Supplementary material for: Investigating the Composition and Metabolic Potential of Microbial Communities in Chocolate Pots Hot Springs
Source: Front Microbiol. 2018 Sep 7;9:2075. doi: 10.3389/fmicb.2018.02075 (PMC6137239; doi:10.3389/fmicb.2018.02075)
Supplement: Supplementary file 1 [file Table_1.PDF]

3. Supplemenary Tables

Supplementary Table 1.

Properties of Chocolate Pots spring water at sampling sites along the flow path.

Sampling Date	Sample Site	Distance from Vent (m)	Temperature (°C)	pH	Fe(II) (mmol L^{-1})[a]
Aug-13	Core 1	0	50.7	5.94	0.103
Aug-13	Core 2	1.0	49.9	6.06	0.090
Aug-13	Core 3	2.1	48.4	6.51	0.052
Aug-13	Core 4	4.1	42.8	7.77	0.006
Aug-13	Core 5	6.8	42.7	7.76	0.008
Aug-13	Core 6	8.2	40.8	7.90	0.004
Aug-13	Entry to Gibbon River	~13	38.1	8.25	0.003
Oct-15	Core 1	0	50.6	5.83	nd[b]
Oct-15	Core 2	1.0	50.2	5.90	nd
Oct-15	Core 3	2.1	47.3	6.46	nd
Oct-15	Core 4	4.1	46.2	6.88	nd
Oct-15	Core 5	6.8	41.9	7.38	nd
Oct-15	Core 6	8.2	40.1	7.56	nd
Oct-15	Vent source	0	51.3	5.76	0.029

[a] *In situ* Fe(II) concentration from the core sampling sites was only measured in 2013. Concentration at the vent source in 2015 was measured at t_0 of the incubation experiments.

[b] Not determined
