# Supplementary material for: Investigating the Composition and Metabolic Potential of Microbial Communities in Chocolate Pots Hot Springs
Source: Front Microbiol. 2018 Sep 7;9:2075. doi: 10.3389/fmicb.2018.02075 (PMC6137239; doi:10.3389/fmicb.2018.02075)
Supplement: Supplementary file 2 [file Table_2.PDF]

Supplementary Table 2.

Phylogenetic assignment and statistics of metagenomic co-assembly of the Chocolate Pots cores.

			Co-assmebly		Core 1		Core 2		Core 3								
MAG#	GC%	MAG size (Mbp)	% Total Reads	Average assembly coverage	% Total Reads[c]	Average normalized coverage[d]	% Total Reads	Average normalized coverage	% Total Reads	Average normalized coverage	% Completeness	% Contamination	% Strain heterogeneity	Concensus Phylogeny	Taxonomic Rank	% Similarity to 16S amplicon library[e]	% Identity to GenBank[f]
0	56.4	6.590	2.32	40.08	0.09	1.10	0.48	4.34	6.04	30.57	96.59	4.2	0	*Deferrisoma camini*	Species	N/A[d]	
1	66.1	2.148	4.08	180.42	0.87	11.10	10.89	159.47			97.47	1.6	0	Euryarchaeota	Phylum	99.3[g]	85.6
2	45.8	3.387	0.08	2.69					0.23	2.26	89.64	4.37	0	*Deferrisoma camini*	Species	N/A	
4	56.1	4.107	0.59	15.96					1.68	14.11	94.94	5.45	0	*Ca.* Nitrospira defluvii	Species	N/A	
6	66.0	4.771	0.19	3.70					0.40	2.08	85.54	8.05	52.63	*Acidobacteriales*	Order	N/A	
7	52.0	2.090	0.07	3.53			0.11	1.67	0.08	1.16	96.45	0	0	*Pelodictyon*	Genus	N/A	
10	57.6	1.183	0.03	2.35					0.06	1.53	55.62	0	0	Acidobacteria	Phylum	N/A	
11	63.1	3.495	0.20	5.64	0.58	4.86					92.59	0.93	0	*Chthonomonas calidirosea*	Species	N/A	
13.2	66.2	5.307	0.40	7.43	0.28	1.61	0.58	3.51	0.31	1.88	95.27	3.51	0	*Ca.* Solibacter usitatus	Species	98.9	87.6
13.3	68.2	4.292	0.39	8.98	0.54	3.64	0.66	4.93			92.05	2.26	27.27	*Ca.* Solibacter	Genus	N/A	
14	27.2	0.880	0.20	25.31	0.66	25.16					89.72	0	0	Archaea	Kingdom	98.9	75.7
15.1	59.7	5.460	0.22	4.25			0.60	3.75			90.61	3.82	20	*Anaerolinea thermophila*	Species	N/A	
15.2	57.6	3.838	0.20	5.89	0.59	4.44	0.07	1.32			99.09	5.64	0	*Anaerolinea thermophila*	Species	N/A	
16	63.7	2.534	0.33	13.60	0.18	2.08	0.76	10.19			89.73	1.56	35.71	*Methyloversatilis*	Genus	N/A	
20	38.3	3.982	0.10	2.61	0.33	2.60					83.44	9.84	0	*Haliangium ochraceum*	Species	N/A	
22	69.7	3.254	0.11	3.41			0.31	3.10			85.54	2.97	0	*Dehalococcoides*	Genus	N/A	
25	53.7	1.754	0.20	13.28					0.57	11.71	93.24	1.35	0	*Simkania negevensis*	Species	N/A	
26	69.7	2.547	0.06	2.36			0.13	1.75			54.92	0.92	0	*Thermomicrobia*	Class	N/A	
27	51.0	1.493	0.03	2.24			0.08	1.83			69.95	8.25	7.14	*Ca.* Caldiarchaeum subterraneum	Species	N/A	
28	60.5	2.665	0.05	2.17			0.14	1.88			63.32	4.39	0	*Nitrospina gracilis*	Species	N/A	
29	58.5	1.715	0.04	2.41	0.13	2.32					71.35	0	0	*Dehalococcoides*	Genus	N/A	
33	55.5	2.631	0.45	20.48	0.12	1.88	1.12	16.74			88.64	2.91	10	*Anaerolinea thermophila*	Species	N/A	
34	52.7	3.054	2.62	93.04	0.18	2.05	7.07	81.33	0.24	4.40	93.97	1.37	0	*Ignavibacteriales*	Order	98.5	84.4
35.1	66.4	4.848	0.28	5.52					0.78	4.85	94.83	0.1	0	*Pirellula staleyi*	Species	99.3	90.2
35.2	65.8	6.133	0.61	10.77					1.74	9.44	99.37	0	0	*Rhodopirellula*	Genus	99.6[g]	88.2
38	45.8	2.948	0.07	2.41	0.22	2.04					83.94	12.99	11.76	*Anaerolinea thermophila*	Species	N/A	
39	55.3	3.982	5.98	153.76	0.49	3.86	1.68	16.74	14.90	117.50	96.17	2.73	0	*Ignavibacteriales*	Order	99.3[g]	82.0
45	33.5	3.122	0.13	5.37	0.44	5.30					91.88	1.71	0	Acidobacteria	Phylum	N/A	
47	53.4	3.003	0.07	2.57					0.20	2.25	74.70	0.93	0	*Chthonomonas calidirosea*	Species	N/A	
48	58.6	1.143	0.04	3.31	0.12	3.19					90.89	0	0	Archaea	Kingdom	N/A	
51	48.0	4.392	0.73	19.90					2.04	16.92	98.52	1.31	0	*Sediminibacterium*	Genus	N/A	
52.2	73.1	1.942	0.24	11.86			0.66	10.82			87.04	1.23	0	*Chthonomonas calidirosea*	Species	N/A	
54	58.5	5.419	0.31	6.21			0.79	5.16			94.44	0.93	0	*Chthonomonas calidirosea*	Species	N/A	
56	60.5	2.851	0.06	2.21			0.13	1.50			50.43	0.91	0	*Anaerolinea thermophila*	Species	N/A	
57.2	42.0	0.670	0.03	4.04	0.08	3.41					52.78	0	0	*Chthonomonas calidirosea*	Species	N/A	
58	59.8	2.700	0.07	2.70	0.12	1.33	0.10	1.28			87.19	0	0	*Dehalococcoides*	Genus	N/A	
59.2	45.0	1.007	0.05	5.86	0.02	1.16			0.11	3.29	74.42	0	0	*Dehalococcoides*	Genus	100.0	94.9
60	62.9	3.820	0.11	3.10					0.28	2.39	78.64	1.92	0	*Anaerolinea thermophila*	Species	N/A	
61	39.9	1.340	0.06	3.45			0.13	2.03			72.06	2.04	0	Bacteria	Kingdom	N/A	
64.1	43.7	1.760	0.10	6.50	0.34	6.42					94.09	1.82	0	*Thermodesulfovibrio*	Genus	N/A	
64.2	45.0	2.728	0.41	18.20	1.36	17.58					94.84	3.55	0	*Desulfobacterium anilini*	Species	N/A	
64.4	44.8	1.613	1.07	78.42	3.10	67.66	0.37	8.83	0.06	1.26	90.91	0	0	*Thermodesulfovibrio*	Genus	N/A	
65.1	48.6	2.440	0.10	4.21	0.15	1.77	0.15	1.93			94.22	0.71	0	*Pseudanabaena*	Genus	N/A	
65.2	47.4	5.389	0.29	6.24					0.80	5.08	98.58	1.18	0	*Oscillatoriales*	Order	98.9	89.5

66	64.5	5.853	0.34	5.87	1.09	5.62					93.52	4.26	0	*Chthonomonas calidirosea*	Species	N/A	
67	58.5	2.322	0.25	11.31	0.23	3.10	0.50	7.66			89.09	1.36	0	*Anaerolinea thermophila*	Species	N/A	
68	37.4	2.768	0.36	16.11	1.05	14.33	0.09	1.03			95.80	2.52	0	*Deferrisoma camini*	Species	N/A	
69.1	68.8	3.161	1.23	38.53	0.20	4.61	3.33	31.94			94.87	2.56	0	*Acidobacteriaceae*	Family	N/A	
69.2	71.2	2.024	0.81	38.41	2.46	34.84	0.20	3.21			95.80	0.42	100	*Deferrisoma camini*	Species	N/A	
70	64.4	3.457	0.79	25.26	2.62	24.92					82.00	0	0	*Anaerolinea thermophila*	Species	N/A	
73	64.0	3.702	0.45	11.88	0.25	2.03	1.01	8.89			96.36	2.27	0	*Anaerolinea thermophila*	Species	N/A	
75	62.3	4.018	0.14	3.52	0.43	3.20					84.91	1.82	0	*Caldilinea aerophila*	Species	N/A	
76	46.4	0.886	0.02	2.39	0.07	2.29					90.51	4.67	0	Archaea	Kingdom	N/A	
79.1	45.1	2.593	0.16	6.97	0.04	1.05	0.35	4.52	0.07	1.02	92.24	2.75	0	*Ignavibacteriales*	Order	99.3	83.4
79.2	45.2	3.662	0.67	20.32	1.79	16.14	0.38	3.82			95.34	2.46	0	*Ignavibacteriales*	Order	N/A	
81	58.1	2.443	0.06	2.48			0.15	2.17			79.68	3.64	12.5	*Thermodesulfovibrio*	Genus	N/A	
84.2	38.8	2.171	0.36	19.96					1.03	17.12	95.10	2.25	0	*Simkania negevensis*	Species	N/A	
87	61.2	2.668	0.07	2.84	0.21	2.59					54.15	1.82	0	*Anaerolinea thermophila*	Species	N/A	
88	66.0	2.945	0.08	2.75			0.22	2.45			77.32	0.84	0	*Deferrisoma camini*	Species	N/A	
89	56.3	3.104	0.54	21.17	0.11	1.27	0.26	3.48	1.18	14.41	86.55	1.82	0	*Anaerolinea thermophila*	Species	N/A	
90.1	70.2	3.122	0.13	4.02					0.32	2.98	80.67	5.91	4	*Frankia*	Genus	N/A	
90.2	72.2	4.251	0.40	9.02			0.23	1.71	0.91	6.33	77.11	5.13	0	*Acidobacteriaceae*	Family	N/A	
94	64.3	2.568	0.09	3.58			0.23	3.13			66.17	2.69	18.18	*Bradyrhizobiaceae*	Family	N/A	
96	36.0	2.017	0.06	3.10	0.19	3.03					89.25	0	0	Bacteria	Kingdom	N/A	
97	72.6	2.385	0.16	6.10			0.35	4.42	0.09	1.09	70.83	0	0	*Dehalococcoides*	Genus	N/A	
98	44.9	1.944	0.04	2.30					0.10	1.70	66.25	1.64	0	*Ignavibacteriales*	Order	N/A	
101.1	42.6	2.987	0.09	3.41					0.26	3.02	98.77	1.97	0	*Chitinophagaceae*	Family	N/A	
101.2	48.1	2.916	0.12	4.25					0.33	3.77	97.62	2.68	0	*Fulvivirga imtechensis*	Species	N/A	
104	66.5	4.007	0.60	15.03	1.70	12.66	0.25	2.12			95.91	3.52	0	*Anaerolinea thermophila*	Species	99.3	87.3
105	72.1	2.990	0.62	19.66					1.75	17.36	90.34	0	0	*Planctomycetaceae*	Family	98.5	82.2
106	60.8	4.418	0.18	3.95	0.59	3.82					95.27	4.84	11.11	*Deltaproteobacteria*	Class	N/A	
108.2	57.0	2.117	0.12	5.77			0.32	4.79			99.07	4.67	0	*Nitrosopumilaceae*	Family	N/A	
108.3	54.7	1.802	0.18	10.86			0.50	9.42			94.82	0	0	*Ca.* Caldiarchaeum subterraneum	Species	100.0	79.9
109	65.9	2.261	1.07	48.61	0.35	5.36	2.59	38.35	0.16	2.39	95.71	0.99	0	*Dehalococcoides*	Genus	N/A	
111	62.9	4.216	0.24	6.34	0.66	4.87	0.12	1.31			90.00	6.09	0	*Anaerolinea thermophila*	Species	N/A	
114	39.0	2.508	0.16	6.70			0.20	2.73	0.21	2.72	95.71	0.24	0	Bacteroidetes	Phylum	N/A	
115	55.2	3.519	0.13	3.86			0.29	2.54			99.80	1.12	0	*Ca.* Endomicrobium	Genus	100.0	85.4
116	60.9	4.803	0.13	2.88					0.36	2.49	79.79	3.36	0	*Deferrisoma camini*	Species	N/A	
119	55.7	4.534	0.43	9.83	0.49	3.28	0.81	6.17			93.52	1.11	0	*Chthonomonas calidirosea*	Species	100.0	81.7
122	64.1	4.634	0.65	12.92					1.83	11.08	93.77	0.93	0	*Chthonomonas calidirosea*	Species	98.2	83.4
123	30.8	2.195	0.05	2.44			0.11	1.70			75.49	1.4	0	*Melioribacter roseus*	Species	N/A	
125	59.7	5.698	0.34	6.98			0.84	5.67			89.81	2.04	0	*Chthonomonas calidirosea*	Species	N/A	
127	72.7	2.103	0.17	7.47			0.34	4.95	0.08	1.12	85.19	1.52	0	*Acidimicrobium ferrooxidans*	Species	N/A	
128.1	59.1	3.395	0.26	7.98			0.03	1.29	0.72	5.84	95.55	8.18	0	*Ca.* Nitrospira defluvii	Species	N/A	
128.2	61.3	3.001	0.32	11.39			0.42	4.98	0.49	5.35	93.18	2.73	0	*Ca.* Nitrospira defluvii	Species	98.9[g]	98.0
128.3	59.7	3.870	0.62	17.05			1.60	14.50	0.15	1.41	94.02	2.56	0	*Acidobacteriaceae*	Family	N/A	
129	61.4	3.394	0.09	2.84			0.24	2.39			93.93	3.23	0	*Planctomycetaceae*	Family	N/A	
133	31.8	3.057	0.23	8.32	0.63	6.72	0.13	1.49			92.31	3.42	0	Acidobacteria	Phylum	N/A	
134	67.7	3.852	0.24	6.71			0.66	6.10			85.51	1.94	0	*Anaeromyxobacter*	Genus	N/A	
135.1	56.9	2.926	0.14	5.14	0.39	3.93	0.08	1.10			69.57	5.54	20	Chloroflexi	Phylum	N/A	
135.2	56.4	4.197	0.53	15.13	1.62	13.19	0.14	1.76			89.60	17.19	29.17	Chloroflexi	Phylum	N/A	
136	65.0	4.506	0.16	3.57	0.39	2.51					90.26	1.55	0	*Caldilinea aerophila*	Species	N/A	
139	41.6	1.681	0.06	4.05			0.16	3.55			95.64	0	0	*Actinobacteria*	Class	N/A	

140	56.2	3.413	0.20	6.29			0.52	5.35			90.91	4.73	0	*Anaerolinea thermophila*	Species	N/A	
141	57.4	1.810	0.05	2.47	0.09	1.48					73.28	0	0	Bacteria	Kingdom	N/A	
145.1	51.1	3.368	0.09	2.76			0.23	2.28			88.58	1.91	25	*Ignavibacteriales*	Order	N/A	
145.2	50.7	2.996	0.18	3.39			0.03	1.20	0.48	4.60	97.52	1.91	0	*Ignavibacteriales*	Order	N/A	
146	65.0	3.501	0.74	23.21	2.46	23.01					87.73	2.73	0	*Anaerolinea thermophila*	Species	99.3[g]	89.7
150	62.3	3.557	0.13	3.46	0.41	3.23					93.52	0	0	*Chthonomonas calidirosea*	Species	N/A	
153	61.2	2.382	0.05	2.21			0.11	1.47			63.96	3.9	0	Chloroflexi	Phylum	N/A	
154.4	38.0	0.826	0.15	20.25			0.00	1.38	0.44	16.64	62.50	0.93	0	Chloroflexi	Phylum	100.0	78.4
157.1	48.6	0.658	0.03	4.48			0.09	4.04			62.16	2.78	0	*Chthonomonas calidirosea*	Species	N/A	
157.2	46.8	0.899	0.05	6.85					0.15	5.51	60.65	0.93	0	*Chthonomonas calidirosea*	Species	99.6	79.0
158.1	63.8	4.390	0.12	2.92					0.33	2.36	89.00	5.11	0	*Planctomycetaceae*	Family	N/A	
158.2	64.5	4.213	0.20	4.80			0.54	3.87			94.59	1.75	0	*Ca.* Solibacter usitatus	Species	N/A	
159	61.9	3.854	0.11	3.01	0.35	2.95					88.06	0.43	100	*Desulfomonile tiedjei*	Species	N/A	
160.2	51.7	0.729	0.02	3.39			0.05	2.43			78.83	1.01	0	*Nitrosopumilaceae*	Family	N/A	
160.3	40.9	0.896	0.12	14.80					0.34	13.13	75.93	0	0	Archaea	Kingdom	N/A	
161	66.0	3.519	0.08	2.33					0.22	2.05	52.92	0	0	*Planctomyces*	Genus	N/A	
162	65.2	4.734	0.46	10.38	0.07	2.36			1.23	6.43	97.27	6.36	0	*Anaerolinea thermophila*	Species	N/A	
163	56.6	1.878	0.05	2.86	0.16	2.74					89.57	0	0	*Dehalococcoides*	Genus	N/A	
164.1	52.6	6.212	0.61	7.84			1.61	6.58			98.22	11.33	0	*Nitrosopumilaceae*	Family	N/A	
164.2	53.5	2.221	1.15	53.68	0.27	3.85	3.03	45.98	0.02	1.05	94.01	1.29	0	*Nitrosopumilaceae*	Family	N/A	
165	55.1	2.411	1.57	77.71	4.45	65.37	0.71	11.55			71.59	0.31	33.33	Chloroflexi	Phylum	97.1	86.9
167	69.7	3.232	0.16	4.99					0.47	4.40	64.80	0.43	0	*Rhodospirillaceae*	Family	N/A	
168	31.9	2.263	0.11	5.33			0.27	4.23			94.76	0.48	0	Bacteroidetes	Phylum	N/A	
171	61.6	1.805	1.34	79.39	0.06	1.07	3.06	59.75	0.70	13.43	95.24	0	0	*Sideroxydans lithotrophicus*	Species	N/A	
172	61.1	2.553	0.08	3.35	0.25	3.06					79.60	0	0	*Anaerolinea thermophila*	Species	N/A	
174	54.3	3.260	0.66	22.86	0.06	1.07	0.44	6.01	1.37	13.72	97.81	0.82	0	*Ignavibacteriales*	Order	N/A	
179	57.1	2.994	0.54	19.64					1.51	17.14	88.18	3.31	0	*Anaerolinea thermophila*	Species	N/A	
181	50.1	2.095	0.12	5.66	0.33	4.47	0.07	1.13			96.55	0	0	Bacteria	Kingdom	N/A	
184	59.3	3.961	0.29	6.87	0.83	5.81					95.29	0	0	*Chthonomonas calidirosea*	Species	99.6	83.8
185	54.5	0.970	0.03	2.77	0.08	2.48					78.89	1.87	0	Archaea	Kingdom	N/A	
186	67.4	4.670	0.55	11.80	0.21	1.32	0.99	7.08	0.39	2.66	94.59	5.07	0	*Pedosphaera parvula*	Species	99.3	91.5
187	60.3	2.243	0.08	3.78	0.26	3.57					94.38	1.12	0	*Ca.* Endomicrobium	Genus	N/A	
189.2	48.0	0.530	0.02	4.12			0.05	3.12			60.53	0	0	*Dehalococcoides*	Genus	N/A	
190	60.5	3.561	0.30	8.65	0.17	1.80	0.62	5.17	0.08	1.23	94.55	7.61	0	*Anaerolinea thermophila*	Species	N/A	
191	60.1	4.924	0.15	3.16	0.17	1.12	0.25	1.82			93.08	0.73	33.33	*Roseiflexus*	Genus	N/A	
194	29.3	2.867	0.50	9.16			1.23	5.96	0.17	1.95	98.54	5.1	0	*Nitrosopumilaceae*	Family	99.6[g]	90.0
196	53.3	2.412	0.10	4.54			0.26	3.38			91.53	0	0	*Thermosipho*	Genus	N/A	
197.1	58.4	5.905	8.78	168.63					24.96	148.24	95.54	1.1	0	*Caldithrix abyssi*	Species	99.6[g]	83.0
200	48.8	2.477	0.07	2.90	0.16	1.96					90.38	0.79	20	*Synechococcus*	Genus	N/A	
202	51.0	2.125	0.27	11.70	0.44	5.70	0.39	5.64			93.10	1.72	0	Bacteria	Kingdom	99.6	81.0
203	64.2	3.665	0.29	8.79	0.96	8.61					85.78	0	0	Chloroflexi	Phylum	99.3	85.0
208	56.4	4.014	0.30	7.87	0.84	6.50	0.13	1.27			96.77	1.29	0	*Desulfomonile tiedjei*	Species	99.6	88.6
209	51.7	2.584	2.33	96.53			1.55	21.99	5.06	64.97	93.72	0.55	0	*Ignavibacteriales*	Order	N/A	
214	68.8	1.851	0.05	2.88	0.14	2.26					64.75	0.85	0	Acidobacteria	Phylum	N/A	
216	64.2	3.319	0.08	2.36			0.15	1.30			65.67	14.21	2.44	*Pedosphaera parvula*	Species	N/A	
220.2	31.2	2.602	0.82	35.05	2.64	32.57	0.10	2.20			93.44	5.19	0	*Ignavibacteriales*	Order	N/A	
220.3	32.9	2.477	1.00	46.41	2.73	37.62	0.51	7.89			88.51	1.09	0	*Ignavibacteriales*	Order	N/A	
221	66.4	2.926	0.18	6.09	0.58	5.94					88.53	0.18	0	*Chloroflexaceae*	Family	100.0	83.0
225.1	56.1	1.643	0.28	19.24	0.60	11.97	0.23	6.32			64.79	0	0	*Thermosipho*	Genus	N/A	

226	54.5	3.835	0.11	2.82	0.18	1.36	0.16	1.37			92.91	3.38	0	*Pedosphaera parvula*	Species	N/A	
227	62.3	3.279	0.33	10.57	0.11	1.38	0.83	8.68			93.97	3.56	0	*Thermodesulfovibrio*	Genus	N/A	
228.1	36.2	0.572	0.04	7.62	0.13	7.41					50.00	7.27	0	*Ca.* Endomicrobium	Genus	78.3	79.3
230.1	36.5	2.814	0.07	2.96	0.19	2.28					87.18	1.4	0	*Thermodesulfovibrio*	Genus	N/A	
232	70.3	2.287	0.12	4.94					0.31	4.10	51.47	0.29	0	*Thermomicrobia*	Class	N/A	
234.2	66.4	2.360	0.43	18.81			0.09	2.69	1.11	14.10	93.73	1.98	0	*Dehalococcoides*	Genus	N/A	
235	30.6	2.766	0.80	32.54	2.66	32.15					95.73	0	0	Acidobacteria	Phylum	N/A	
236	67.6	2.715	0.46	15.67	1.14	11.47	0.35	3.96			94.44	2.14	33.33	Acidobacteria	Phylum	98.9	98.3
237	43.5	3.870	0.22	5.96	0.72	5.85					96.13	3.55	0	*Desulfomonile tiedjei*	Species	N/A	
238	48.0	3.249	0.20	6.72	0.68	6.62					91.97	3.42	0	Acidobacteria	Phylum	N/A	
241	61.5	3.660	0.16	4.12			0.23	1.97	0.14	1.17	93.28	3.36	0	*Deferrisoma camini*	Species	N/A	
244	60.7	2.139	0.04	2.22					0.08	1.21	59.13	0.19	0	*Dehalococcoides*	Genus	N/A	
245.1	70.1	3.183	0.27	8.53			0.68	7.24			81.77	0.93	0	*Chthonomonas calidirosea*	Species	N/A	
245.2	69.9	2.805	0.57	20.44			1.50	18.00	0.11	1.18	93.10	8.15	31.25	*Chthonomonas calidirosea*	Species	N/A	
245.3	68.1	2.502	0.73	30.55			2.01	27.49	0.05	1.04	92.59	0.93	0	*Chthonomonas calidirosea*	Species	99.3	83.0
246	66.5	4.489	0.16	3.63					0.43	2.95	73.64	5.91	8.33	*Bradyrhizobiaceae*	Family	N/A	
247	61.6	4.322	0.12	2.80	0.37	2.60					82.65	2.93	0	*Deltaproteobacteria*	Class	N/A	
249	43.1	1.122	0.10	9.54	0.33	9.06					74.46	5.61	57.14	Archaea	Kingdom	99.6	76.6
250.1	48.6	1.951	0.15	8.68	0.31	5.44	0.13	2.66			94.55	1.82	0	*Thermodesulfovibrio*	Genus	N/A	
250.2	49.3	2.026	0.90	49.53			2.54	45.99			95.85	1.82	0	*Ca.* Nitrospira defluvii	Species	N/A	
251	68.7	2.744	1.47	52.80	0.17	1.88	2.85	34.07	1.18	13.23	98.90	1.1	0	*Gemmatimonas aurantiaca*	Species	N/A	
252.1	30.7	1.759	0.05	3.30	0.18	3.21					91.61	0	0	*Ca. Cloacamonas acidaminovorans*	Species	99.4	79.6
252.2	36.8	2.347	0.08	3.30	0.26	3.40					91.28	2.33	0	Acidobacteria	Phylum	N/A	
254	68.5	3.539	0.39	10.32			0.27	2.44	0.76	6.33	90.43	1.75	0	*Sutterella wadsworthensis*	Species	97.4	93.6
255	62.1	1.920	0.16	8.50	0.53	8.39					98.40	0.8	0	Euryarchaeota	Phylum	99.6	87.8
**sum**[a]			72.72		56.29		73.03		82.66								
**avg**[b]				*15.09*		*7.94*		*9.75*		*11.27*							

[a] Total percentage of reads mapping to MAGs in a given metagenomic assembly

[b] Average read coverage of the contigs in each MAG

[c] Calculated as the percent of reads mapped to a given MAG out of the total number of normalized reads for an individual core library

[d] MAGs with a calculated coverage of <1.0 are considered to be absent from a metagenomic library for an individual core, and not listed

[e] Alignment between 16S rRNA gene sequences from the metagenomic co-assembly and the 16S rRNA gene amplicon library

[f] %Identity of 16S rRNA gene sequences from the metagenomic library aligned to the NCBI GenBank database

[g] Not applicable; no significant alignment to 16S rRNA gene amplicon library, or metagenomic 16S rRNA gene sequence on unbinned contig

[h] >1% OTU read abundance in 16S rRNA gene amplicon library
