# Supplementary material for: Investigating the Composition and Metabolic Potential of Microbial Communities in Chocolate Pots Hot Springs
Source: Front Microbiol. 2018 Sep 7;9:2075. doi: 10.3389/fmicb.2018.02075 (PMC6137239; doi:10.3389/fmicb.2018.02075)
Supplement: Supplementary file 3 [file Table_3.PDF]

Supplementary Table 3.

Phylogenetic assignment and statistics of metagenomic assembly of the Chocolate Pots vent pool water column.

MAG#	GC %	MAG size (Mbp)	% Total Reads[c]	Average assembly coverage	% Completeness	% Contamination	% Strain heterogeneity	Concensus Phylogeny	Taxonomic rank	% Similarity to 16S amplicon library[d]	% Identity to GenBank[e]
2	43.7	1.217	0.16	5.53	87.20	3.2	50	*Methanobacteriaceae*	Family	N/A[f]	
3	70.8	2.374	0.67	8.56	96.64	2.1	0	*Deferrisoma camini*	Species	100	87.0
7	31.2	2.388	0.87	15.88	92.90	0.55	0	*Ignavibacteriales*	Order	100	85.5
9	68.1	1.955	0.22	4.25	56.01	6.27	0	*Chthonomonas calidirosea*	Species	N/A	
11	66.3	4.226	1.11	8.75	97.27	3.36	0	*Anaerolinea thermophila*	Species	100	88.4
12	36.2	2.670	0.68	9.81	96.72	4.37	44.44	*Ignavibacteriales*	Order	100	86.8
13	68.0	1.910	0.37	6.23	79.72	2.14	33.33	*Thermoanaerobaculum*	Genus	100	99.0
14	33.3	2.007	0.48	11.97	98.19	0.88	0	*Calditerrivibrio nitroreducens*	Species	N/A	
16	38.7	2.507	3.49	60.66	95.79	0.55	0	*Ignavibacteriales*	Order	100[g]	84.4
17	60.0	2.869	1.53	20.17	98.10	1.71	0	*Sideroxydans lithotrophicus*	Species	100[g]	95.2
20	32.3	2.163	0.29	6.04	50.68	12.93	0	*Ca.* Endomicrobium	Genus	N/A	
21	38.0	1.188	0.14	5.13	75.62	1.7	50	*Planctomycetaceae*	Family	98.8	78.1
22	49.0	3.076	10.41	148.08	98.36	1.91	0	*Pelodictyon*	Genus	100	86.3
25	55.2	0.791	0.10	4.99	61.41	0.56	0	*Ca.* Endomicrobium	Genus	100	84.4
31	39.2	1.234	0.18	6.08	60.34	7.08	0	*Deinococci*	Class	100	92.5
33	63.8	2.709	1.54	19.42	93.47	1.39	66.67	*Methyloversatilis*	Genus	N/A	
34	60.3	5.659	7.71	59.16	99.69	2.36	75	*Roseiflexus*	Genus	N/A	
36	37.3	2.058	0.34	7.18	94.91	3.36	25	*Deferrisoma camini*	Species	100	85.0
37	69.8	2.211	0.34	5.60	80.46	4.68	42.86	*Dehalococcoides*	Genus	99.6	85.4
38	30.5	2.019	0.24	5.15	70.59	0	0	*Leptonema illini*	Species	N/A	
42	34.1	0.583	1.51	120.02	63.92	0	0	Bacteria	Kingdom	100	76.8
46	58.3	2.555	1.30	20.85	95.45	0.91	0	*Anaerolinea thermophila*	Species	N/A	
49	31.8	1.419	2.04	127.06	82.10	3.96	12.5	*Thermococcaceae*	Family	N/A	
51	39.7	1.559	0.18	5.18	71.13	1.04	100	*Thermodesulfovibrio*	Genus	100	85.6
52	37.7	1.328	0.23	7.38	92.37	0	0	*Caldisericum exile*	Species	83.1	98.3
53	42.8	1.760	0.48	11.64	97.27	0.91	0	*Thermodesulfovibrio*	Genus	N/A	
54	47.4	3.227	3.26	49.63	98.91	1.91	0	*Chloroherpeton thalassium*	Species	100[g]	86.6
56	36.6	1.883	0.68	15.21	89.33	2.25	50	*Ca.* Endomicrobium	Genus	100	80.3
59	54.9	4.124	2.64	24.71	87.46	4.62	33.33	*Anaerolineaceae*	Family	100[g]	87.2
63	29.5	0.758	0.11	6.08	85.59	0.93	100	Archaea	Kingdom	82.4	77.7
64	48.3	2.661	12.48	177.95	96.58	0.24	0	*Pseudanabaena*	Genus	100[g]	90.3
67	42.0	1.986	0.70	15.41	95.61	23.44	52.38	*Thermodesulfovibrio*	Genus	N/A	
69	58.8	2.774	0.42	6.82	99.12	0	0	*Synechococcus*	Genus	N/A	
70	34.5	0.599	0.34	24.44	71.84	0	0	*Bacteria*	Kingdom	N/A	
73	39.5	1.557	0.96	29.22	93.75	0	0	*Ca. Endomicrobium*	Genus	100	83.2
74	45.9	3.179	2.14	26.58	97.27	6.36	25	*Thermodesulfovibrio*	Genus	98.8[g]	88.9
75	56.8	1.891	0.21	4.78	76.03	1.46	60	Bacteroidetes	Phylum	100	80.8
77	33.0	1.875	0.27	5.56	65.43	1.73	50	*Ignavibacteriales*	Order	N/A	
78	44.7	1.767	2.47	65.80	94.55	0.91	0	*Thermodesulfovibrio*	Genus	N/A	
79	60.9	1.152	0.11	4.19	54.57	0.03	100	*Uliginosibacterium gangwonense*	Species	100	95.5
80	44.8	2.417	0.30	5.65	91.39	0.88	33.33	*Desulfobacteraceae*	Family	N/A	
81.1	29.9	0.991	10.46	493.44	89.10	3.74	0	Archaea	Kingdom	100	76.5
81.2	31.7	0.928	21.87	1092.94	73.49	1.49	0	*Thermococcaceae*	Family	100	76.6
**sum[a]**			95.99								
**avg[b]**				*28.61*							

[a] Total percentage of reads mapping to MAGs in the metagenomic assembly
[b] Average read coverage of the contigs in each MAG
[c] Calculated as the percent of reads mapped to a given MAG out of the total number reads for the metagenomic library
[d] Alignment between 16S rRNA gene sequences from the metagenomic assembly and the 16S rRNA gene amplicon library
[e] %Identity of 16S rRNA gene sequences from the metagenomic library aligned to the NCBI GenBank database
[f] Not applicable; no significant alignment to 16S rRNA gene amplicon library, or metagenomic 16S rRNA gene sequence on unbinned condig
[g] >1% OTU read abundance in 16S rRNA gene amplicon library
[a] Total percentage of reads mapping to MAGs in the metagenomic assembly
[b] Average read coverage of the contigs in each MAG
