# Supplementary material for: Investigating the Composition and Metabolic Potential of Microbial Communities in Chocolate Pots Hot Springs
Source: Front Microbiol. 2018 Sep 7;9:2075. doi: 10.3389/fmicb.2018.02075 (PMC6137239; doi:10.3389/fmicb.2018.02075)
Supplement: Supplementary file 7 [file Data_Sheet_4.PDF]

SUPPLEMENTARY MATERIAL

Supplementary Text 1.1 (Supplementary Materials and Methods)
Supplementary Text 1.2 (Supplementary Results and Discussion)
Supplementary Text 1.3 (Supplementary References)
Supplementary Figures 2.1 (Visualization of Chocolate Pots cores metagenomic co-assembly statistics)
Supplementary Figure 2.2 (Visualization of Chocolate Pots vent pool water column metagenomic assembly statistics)
Supplementary Figure 2.3 (Presence or absence of metabolic pathways of interest in the Chocolate Pots cores metagenomic co-assembly)
Supplementary Figure 2.4 (Presence or absence of metabolic pathways of interest in the Chocolate Pots vent pool water column metagenomic assembly)
Supplementary Table 3.1 (Properties of Chocolate Pots spring water at sampling sites along flow path)
Supplementary Table 3.2 (Phylogenetic assignment and statistics of metagenomic co-assembly of the Chocolate Pots cores)
Supplementary Table 3.3 (Phylogenetic assignment and statistics of metagenomic assembly of the Chocolate Pots vent pool water column)
Supplementary Data 4.1 (FASTA file containing the 16S rRNA gene amplicon library for the Chocolate Pots cores samples)
Supplementary Data 4.2 (Raw OTU table output from QIIME corresponding to the Chocolate Pots cores 16S library)
Supplementary Data 4.3 (FASTA file containing the 16S rRNA gene amplicon library for the Chocolate Pots vent pool water column)
Supplementary Data 4.4 (Raw OTU table output from QIIME corresponding to the Chocolate Pots vent pool water column 16S library)

1. Supplementary Text

1.1 Supplementary Materials and Methods

The four CO_2 fixation pathways searched for within the metagenomic assemblies were positively identified within a metagenome-assembled genome (MAG) if it encoded all genes predicted to be involved in the pathway. The four CO_2 fixation pathways investigated were: the reductive pentose phosphate cycle [Calvin-Benson-Bassham (CBB)], reductive tricarboxylic acid cycle (rTCA), reductive acetyl-CoA pathway [Wood-Ljungdahl (WL)], and 3-hydroxypropionate (3HP) bicycle.

Calvin-Benson-Bassham cycle. CBB genes in either metagenomic assembly were identified using the Function Search option in IMG/M ER using Enzyme Commission (EC, http://enzyme.expasy.org/) numbers for each enzyme involved in the pathway. The enzyme ribulose bisphosphate carboxylase/oxidase (RuBisCO) is involved in the first step of the CBB cycle and was the basis for determining whether or not a member of the microbial community, represented by a metagenomic MAG, was putatively capable of carbon fixation via the CBB pathway. Putative RuBisCO-encoding archaeal MAGs were investigated for an alternative enzyme for regenerating ribulose 1,5-bisphosphate (RuBP): thiazole-adenylate synthase (TAS) (Finn and Tabita, 2004;Tabita et al., 2008). Additionally, some Archaea can use RuBisCO as part of the ribulose monophosphate (RuMP) pathway (Kono et al., 2017). Key enzymes in this pathway, D-arabino-3-hexulose-6-phosphate synthase and phospho-3-hexuloisomerase, were searched for in RuBisCO-containing archaeal MAGs also. MAGs which did not encode proteins identified as sedoheptulose-1,7-bisphosphatase (SBPase, EC:3.1.3.37) were investigated for the presence of genes encoding potential bifunctional fructose-1,6-bisphosphatase (FBPase, EC:3.1.3.11) (Jiang et al., 2012), or fructose-1,6-bisphosphate aldolase (FBP aldolase, EC:4.1.2.13) (Say and Fuchs, 2010;Du et al., 2011). MAGs were only considered to encode a full CBB pathway if genes encoding all enzymes (or bifunctional variants) were identified. If all key enzymes (e.g. RuBisCO) were coded for in a MAG, and no more than one gene predicted in the pathway was undetected, these were considered partially complete. Despite coding for essential proteins (i.e. RuBisCO) MAGs that did not encode two or more proteins in the CBB pathway were considered too incomplete for further study.

Wood-Ljungdahl pathway. Genes involved in the WL pathway were identified in the metagenomic assemblies using the EC numbers for each enzyme involved in the pathway. There are two key enzymes in the pathway, carbon monoxide dehydrogenase (EC:1.2.7.4, and EC:1.2.99.2) and CO-methylating acetyl-CoA synthase (EC:2.3.1.169). MAGs that did not encode either of these key enzymes were considered too incomplete to investigate further. Despite prior identification of organisms capable of utilizing the WL pathway for CO_2 fixation that do not encode a complete set of proteins, as identified by KEGG (Matschiavelli et al., 2012), in this study MAGs were only considered to encode a complete WL pathway if all of the predicted protein coding genes were present.

Reductive tricarboxylic acid cycle. Genes coding for enzymes involved in the rTCA pathway were identified in the metagenomic assemblies using the EC numbers for each enzyme involved in the pathway. There are three enzymes unique to this pathway

that differentiate it from the TCA cycle: fumarate reductase (EC:1.3.5.4), 2-oxoglutarate synthase (EC:1.2.7.3), and the citrate-cleaving reaction which can occur as a one- or two-step reaction (Hügler and Sievert, 2011). Citrate can be cleaved into oxaloacetate and acetyl-CoA by ATP citrate lyase (EC:2.3.3.8), or citryl-CoA synthetase (CCS, EC:6.2.1.18) and citryl-CoA lyase (CCL, EC:4.1.3.34) with citryl-CoA as an intermediate (Aoshima et al., 2004;Aoshima, 2007). MAGs were only considered to contain a complete rTCA cycle if genes encoding the aforementioned unidirectional enzymes were detected, as well as the genes encoding enzymes that catalyze the reversible reactions in the pathway.

3-hydroxypropionate bicycle. Genes encoding proteins involved in the 3HP pathway were identified in the metagenomic assemblies using EC numbers for each enzyme involved in the pathway. The three key marker proteins from the 3HP pathway, first identified in *Chloroflexus aurantiacus*, are propionyl-CoA/3-hydroxypropionyl-CoA synthetase (EC:6.2.1.17/EC:6.2.1.36), malyl-CoA/mesaconyl-C1-CoA/citramalyl-CoA (MMC) lyase (EC:4.2.3.25/EC:4.2.3.24/EC:4.2.1.148), and malonyl-CoA reductase (EC:1.2.1.75) (Strauss and Fuchs, 1993;Alber and Fuchs, 2002;Hügler et al., 2002;Zarzycki et al., 2008). MAGs were considered to contain a full 3HP pathway if all marker genes were identified. MAGs that did not encode any of the key marker genes were considered too incomplete for further study.

1.2 Supplementary Results and Discussion

Statistics of CP sediment core metagenomes and MAGs. Paired-end 2x100 bp Illumina HiSeq 2000 shotgun metagenomic sequencing produced a total of 103840982, 110482524, and 116861176 reads for cores 1, 2, and 3, respectively. The combined metagenomic assembly (co-assembly) of the cores contained 331184682 reads assembled into 1712324 contigs with an N50 of 3198 bp, and an average length of 938 bp. The CONCOCT binning algorithm identified 256 MAGs; 37 of these were composite MAGs which were manually split based on %GC and coverage into 82 MAGs (Supplementary Figure 1). MAGs that were only partially complete (<50% completeness) or very highly contaminated (>15% contamination) were removed before further analysis (n=134). The remaining 167 high-quality MAGs contained 72.7% of all reads mapped to the metagenomic co-assembly. MAGs had an average completeness of 86.2% ± 12.5%, and an average contamination of 2.4% ± 2.8% (Supplementary Table 2).

Statistics of CP vent pool water column metagenome and MAGs. Paired-end 2x250 Illumina HiSeq 2500 Rapid shotgun metagenomic sequencing produced a total of 48106126 reads assembled into 281407 contigs with an N50 of 1361 bp, and an average length of 1107 bp. The CONCOCT binning algorithm identified 82 MAGs (Supplementary Figure 2); one composite MAG was split in two. MAGs that were only partially complete (<50% completeness) or very highly contaminated (>15% contamination) were removed before further analysis (n=40). The remaining 43 high-quality MAGs contained 96.0% of all reads mapped to the metagenomic assembly. MAGs had an average completeness of 84.8% ± 14.4%, and an average contamination of 2.7% ± 4.1% (Supplementary Table 3). The coverage of the two extremely high-coverage MAGs (ca. 1093x and 493x coverage for *Thermococcaceae* and Archaea, respectively)

was not included in the calculation of average coverage for the metagenomic assembly, due to their extremely high coverage.

1.3 Supplementary References

Alber, B.E., and Fuchs, G. (2002). Propionyl-Coenzyme A Synthase from *Chloroflexus aurantiacus*, a Key Enzyme of the 3-Hydroxypropionate Cycle for Autotrophic CO_2 Fixation. *J Biol Chem* 277**,** 12137-12143. doi: 10.1074/jbc.M110802200

Albertsen, M., Hugenholtz, P., Skarshewski, A., Nielsen, K.R.L., Tyson, G.W., and Nielsen, P.H. (2013). Genome sequences of rare, uncultured bacteria obtained by differential coverage binning of multiple metagenomes. *Nat Biotechnol* 31**,** 533-538. doi: 10.1038/nbt.2579

Allen, E.T., and Day, A.L. (1935). *Hot Springs of the Yellowstone National Park.* Carnegie Institution of Washington.

Alneberg, J., Bjarnason, B.S.R., De Bruijn, I., Schirmer, M., Quick, J., Ijaz, U.Z., Lahti, L., Loman, N.J., Andersson, A.F., and Quince, C. (2014). Binning metagenomic contigs by coverage and composition. *Nat Methods* 11**,** 1144-1146. doi: 10.1038/nmeth.3103

Aoshima, M. (2007). Novel enzyme reactions related to the tricarboxylic acid cycle: phylogenetic/functional implications and biotechnological applications. *Appl Microbiol Biotechnol* 75**,** 249-255. doi: 10.1007/s00253-007-0893-0

Aoshima, M., Ishii, M., and Igarashi, Y. (2004). A novel enzyme, citryl-CoA lyase, catalysing the second step of the citrate cleavage reaction in *Hydrogenobacter thermophilus* TK-6. *Mol Microbiol* 52**,** 763-770. doi: 10.1111/j.1365-2958.2004.04010.x

Arvidson, R.E., Squyres, S.W., Bell, J.F., 3rd, Catalano, J.G., Clark, B.C., Crumpler, L.S., De Souza, P.A., Jr., Fairen, A.G., Farrand, W.H., Fox, V.K., Gellert, R., Ghosh, A., Golombek, M.P., Grotzinger, J.P., Guinness, E.A., Herkenhoff, K.E., Jolliff, B.L., Knoll, A.H., Li, R., Mclennan, S.M., Ming, D.W., Mittlefehldt, D.W., Moore, J.M., Morris, R.V., Murchie, S.L., Parker, T.J., Paulsen, G., Rice, J.W., Ruff, S.W., Smith, M.D., and Wolff, M.J. (2014). Ancient aqueous environments at Endeavour Crater, Mars. *Science* 343**,** 8. doi: 10.1126/science.1248097

Bankevich, A., Nurk, S., Antipov, D., Gurevich, A.A., Dvorkin, M., Kulikov, A.S., Lesin, V.M., Nikolenko, S.I., Pham, S., Prjibelski, A.D., Pyshkin, A.V., Sirotkin, A.V., Vyahhi, N., Tesler, G., Alekseyev, M.A., and Pevzner, P.A. (2012). SPAdes: a new genome assembly algorithm and its applications to single-cell sequencing. *J Comput Biol* 19**,** 455-477. doi: 10.1089/cmb.2012.0021

Blöthe, M., and Roden, E.E. (2009). Microbial iron redox cycling in a circumneutral-pH groundwater seep. *Appl Environ Microbiol* 75**,** 468-473. doi: 10.1128/AEM.01817-08

Brysch, K., Schneider, C., Fuchs, G., and Widdel, F. (1987). Lithoautotrophic growth of sulfate-reducing bacteria, and description of *Desulfobacterium autotrophicum* gen. nov., sp. nov. *Arch Microbiol* 148**,** 264-274. doi:

Camacho, A., Walter, X.A., Picazo, A., and Zopfi, J. (2017). Photoferrotrophy: Remains of an Ancient Photosynthesis in Modern Environments. *Front Microbiol* 8**,** 323. doi: 10.3389/fmicb.2017.00323

Can, M., Armstrong, F.A., and Ragsdale, S.W. (2014). Structure, function, and mechanism of the nickel metalloenzymes, CO dehydrogenase, and acetyl-CoA synthase. *Chem Rev* 114**,** 4149-4174. doi: 10.1021/cr400461p

Canfield, D.E. (2005). The early history of atmospheric oxygen: Homage to Robert M. Garrels. *Ann Rev Earth Planet Sci* 33**,** 1-36. doi: 10.1146/annurev.earth.33.092203.122711

Caporaso, J.G., Lauber, C.L., Walters, W.A., Berg-Lyons, D., Lozupone, C.A., Turnbaugh, P.J., Fierer, N., and Knight, R. (2011). Global patterns of 16S rRNA diversity at a depth of millions of sequences per sample. *Proc Natl Acad Sci U S A* 108 Suppl 1**,** 4516-4522. doi: 10.1073/pnas.1000080107

Chin, K.-J., Lukow, T., Stubner, S., and Conrad, R. (1999). Structure and function of the methanogenic archaeal community in stable cellulose-degrading enrichment cultures at two different temperatures (15 and 30°C). *FEMS Microbiol Ecol* 30**,** 313-326. doi:

Coates, J.D., Ellis, D.J., Gaw, C.V., and Lovley, D.R. (1999). *Geothrix fermentans* gen. nov., sp. nov., a novel Fe(III)-reducing bacterium from a hydrocarbon-contaminated aquifer. *Int J Syst Bacteriol* 49**,** 1615-1622. doi:

Colman, D.R., Feyhl-Buska, J., Robinson, K.J., Fecteau, K.M., Xu, H., Shock, E.L., and Boyd, E.S. (2016). Ecological differentiation in planktonic and sediment-associated chemotrophic microbial populations in Yellowstone hot springs. *FEMS Microbiol Ecol* 92. doi: 10.1093/femsec/fiw137

Crowe, S.A., Jones, C., Katsev, S., Magen, C., O'neill, A.H., Sturm, A., Canfield, D.E., Haffner, G.D., Mucci, A., Sundby, B., and Fowle, D.A. (2008). Photoferrotrophs thrive in an Archean Ocean analogue. *Proc Natl Acad Sci U S A* 105**,** 15938-15943. doi: 10.1073/pnas.0805313105

Du, J., Say, R.F., Lü, W., Fuchs, G., and Einsle, O. (2011). Active-site remodelling in the bifunctional fructose-1,6-bisphosphate aldolase/phosphatase. *Nature* 478**,** 534-537. doi: 10.1038/nature10458

Emerson, D. (2000). "Microbial Oxidation of Fe(II) and Mn(II) at Circumneutral pH," in *Environmental Microbe-Metal Interactions,* ed. D.R. Lovley. (Washington, D.C.: ASM Press), 31-52.

Emerson, D., Field, E.K., Chertkov, O., Davenport, K.W., Goodwin, L., Munk, C., Nolan, M., and Woyke, T. (2013). Comparative genomics of freshwater Fe-oxidizing bacteria: implications for physiology, ecology, and systematics. *Front Microbiol* 4**,** 254. doi: 10.3389/fmicb.2013.00254

Emerson, D., Fleming, E.J., and Mcbeth, J.M. (2010). Iron-oxidizing bacteria: an environmental and genomic perspective. *Ann Rev Microbiol* 64**,** 561-583. doi: 10.1146/annurev.micro.112408.134208

Emerson, D., and Revsbech, N.P. (1994). Investigation of an Iron-Oxidizing Microbial Mat Community Located near Aarhus, Denmark: Laboratory Studies. *Appl Environ Microbiol* 60**,** 4032-4038. doi:

Emerson, D., and Weiss, J.V. (2004). Bacterial Iron Oxidation in Circumneutral Freshwater Habitats: Findings from the Field and the Laboratory. *Geomicrobiol J* 21**,** 405-414. doi: 10.1080/01490450490485881

Finn, M.W., and Tabita, F.R. (2004). Modified Pathway To Synthesize Ribulose 1,5-Bisphosphate in Methanogenic Archaea. *J Bacteriol* 186**,** 6360-6366. doi: 10.1128/JB.186.19.6360-6366.2004

Finn, R.D., Clements, J., and Eddy, S.R. (2011). HMMER web server: interactive sequence similarity searching. *Nucleic Acids Res* 39**,** W29-37. doi: 10.1093/nar/gkr367

Fortney, N.W., He, S., Converse, B.J., Beard, B.L., Johnson, C.M., Boyd, E.S., and Roden, E.E. (2016). Microbial Fe(III) oxide reduction potential in Chocolate Pots hot spring, Yellowstone National Park. *Geobiology* 14**,** 255-275. doi: 10.1111/gbi.12173

Fortney, N.W., He, S., Kulkarni, A., Friedrich, M.W., Holz, C., Boyd, E.S., and Roden, E.E. (2018). Stable isotope probing of microbial iron reduction in Chocolate Pots hot spring, Yellowstone National Park. *Appl Environ Microbiol*. doi: 10.1128/AEM.02894-17

Frank, Y.A., Kadnikov, V.V., Lukina, A.P., Banks, D., Beletsky, A.V., Mardanov, A.V., Sen'kina, E.I., Avakyan, M.R., Karnachuk, O.V., and Ravin, N.V. (2016). Characterization and Genome Analysis of the First Facultatively Alkaliphilic *Thermodesulfovibrio* Isolated from the Deep Terrestrial Subsurface. *Front Microbiol* 7**,** 2000. doi: 10.3389/fmicb.2016.02000

Frigaard, N.-U., and Bryant, D.A. (2008). "Genomic and Evolutionary Perspectives on Sulfur Metabolism in Green Sulfur Bacteria," in *Microbial Sulfur Metabolism,* eds. C. Dahl & C.G. Friedrich. (Berlin: Springer-Verlag), 60-76.

Haaijer, S.C.M., Harhangi, H.R., Meijerink, B.B., Strous, M., Pol, A., Smolders, A.J., Verwegen, K., Jetten, M.S.M., and Op Den Camp, H.J.M. (2008). Bacteria associated with iron seeps in a sulfur-rich, neutral pH, freshwater ecosystem. *ISME J* 2**,** 1231-1242. doi: 10.1038/ismej.2008.75

Hafenbradl, D., Keller, M., Dirmeier, R., Rachel, R., Roßnagel, P., Burggraf, S., Huber, H., and Stetter, K.O. (1996). *Ferroglobus placidus* gen. nov., sp. nov., a novel hyperthermophilic archaeum that oxidizes Fe^{2+} at neutral pH under anoxic conditions. *Arch Microbiol* 16**,** 308-314. doi:

Hartshorne, R.S., Reardon, C.L., Ross, D., Nuester, J., Clarke, T.A., Gates, A.J., Mills, P.C., Fredrickson, J.K., Zachara, J.M., Shi, L., Beliaev, A.S., Marshall, M.J., Tien, M., Brantley, S., Butt, J.N., and Richardson, D.J. (2009). Characterization of an electron conduit between bacteria and the extracellular environment. *Proc Natl Acad Sci U S A* 106**,** 22169-22174. doi: 10.1073/pnas.0900086106

Hattori, S., Galushko, A.S., Kamagata, Y., and Schink, B. (2005). Operation of the CO dehydrogenase/acetyl coenzyme A pathway in both acetate oxidation and acetate formation by the syntrophically acetate-oxidizing bacterium *Thermacetogenium phaeum*. *J Bacteriol* 187**,** 3471-3476. doi: 10.1128/JB.187.10.3471-3476.2005

Hegler, F., Lösekann-Behrens, T., Hanselmann, K., Behrens, S., and Kappler, A. (2012). Influence of Seasonal and Geochemical Changes on the Geomicrobiology of an Iron Carbonate Mineral Water Spring. *Appl Environ Microbiol* 78**,** 7185-7196. doi: 10.1128/AEM.01440-12

Henry, E.A., Devereux, R., Maki, J.S., Gilmour, C.C., Woese, C.R., Mandelco, L., Schauder, R., Remen, C.C., and Mitchell, R. (1994). Characterization of a new thermophilic sulfate-reducing bacterium *Thermodesulfovibrio yellowstonii*, gen.

nov. and sp. nov.: its phylogenetic relationship to *Thermodesulfobacterium commune* and their origins deep within the bacterial domain. *Arch Microbiol* 161**,** 62-69. doi:

Hügler, M., Menendez, C., Schägger, H., and Fuchs, G. (2002). Malonyl-Coenzyme A Reductase from *Chloroflexus aurantiacus*, a Key Enzyme of the 3-Hydroxypropionate Cycle for Autotrophic CO_2 Fixation. *J Bacteriol* 184**,** 2404-2410. doi: 10.1128/jb.184.9.2404-2410.2002

Hügler, M., and Sievert, S.M. (2011). Beyond the Calvin Cycle: Autotrophic Carbon Fixation in the Ocean. *Annu Rev Mar Sci* 3. doi: 10.1146/annurev-marine-120709-142712

Hügler, M., Wirsen, C.O., Fuchs, G., Taylor, C.D., and Sievert, S.M. (2005). Evidence for Autotrophic CO_2 Fixation via the Reductive Tricarboxylic Acid Cycle by Members of the ε Subdivision of Proteobacteria. *J Bacteriol* 187**,** 3020-3027. doi: 10.1128/JB.187.9.3020-3027.2005

Huson, D.H., Auch, A.F., Qi, J., and Schuster, S.C. (2007). MEGAN analysis of metagenomic data. *Genome Res* 17**,** 377-386. doi: 10.1101/gr.5969107

Huson, D.H., and Scornavacca, C. (2012). Dendroscope 3: an interactive tool for rooted phylogenetic trees and networks. *Syst Biol* 61**,** 1061-1067. doi: 10.1093/sysbio/sys062

Hyatt, D., Chen, G.-L., Locascio, P.F., Land, M.L., Larimer, F.W., and Hauser, L.J. (2010). Prodigal: prokaryotic gene recognition and translation initiation site identification. *BMC bioinformatics* 11**,** 119. doi:

Iino, T., Mori, K., Uchino, Y., Nakagawa, T., Harayama, S., and Suzuki, K. (2010). *Ignavibacterium album* gen. nov., sp. nov., a moderately thermophilic anaerobic bacterium isolated from microbial mats at a terrestrial hot spring and proposal of *Ignavibacteria* classis nov., for a novel lineage at the periphery of green sulfur bacteria. *Int J Syst Evol Micr* 60**,** 1376-1382. doi: 10.1099/ijs.0.012484-0

Ilbert, M., and Bonnefoy, V. (2013). Insight into the evolution of the iron oxidation pathways. *Biochim Biophys Acta* 1827**,** 161-175. doi: 10.1016/j.bbabio.2012.10.001

Imhoff, J.F. (2003). Phylogenetic taxonomy of the family *Chlorobiaceae* on the basis of 16S rRNA and *fmo* (Fenna-Matthews-Olson protein) gene sequences. *Int J Syst Evol Micr* 53**,** 941-951. doi: 10.1099/ijs.0.02403-0

Jiang, Y.-H., Wang, D.-Y., and Wen, J.-F. (2012). The independent prokaryotic origins of eukaryotic fructose-1, 6-bisphosphatase and sedoheptulose-1, 7-bisphosphatase and the implications of their origins for the evolution of eukaryotic Calvin cycle. *BMC Evol Biol* 12**,** 208. doi:

Klatt, C.G., Bryant, D.A., and Ward, D.M. (2007). Comparative genomics provides evidence for the 3-hydroxypropionate autotrophic pathway in filamentous anoxygenic phototrophic bacteria and in hot spring microbial mats. *Environ Microbiol* 9**,** 2067-2078. doi: 10.1111/j.1462-2920.2007.01323.x

Klatt, C.G., Inskeep, W.P., Herrgard, M.J., Jay, Z.J., Rusch, D.B., Tringe, S.G., Parenteau, M.N., Ward, D.M., Boomer, S.M., Bryant, D.A., and Miller, S.R. (2013). Community structure and function of high-temperature chlorophototrophic microbial mats inhabiting diverse geothermal environments. *Front Microbiol* 4**,** 106. doi: 10.3389/fmicb.2013.00106

Kono, T., Mehrotra, S., Endo, C., Kizu, N., Matusda, M., Kimura, H., Mizohata, E., Inoue, T., Hasunuma, T., Yokota, A., Matsumura, H., and Ashida, H. (2017). A RuBisCO-mediated carbon metabolic pathway in methanogenic archaea. *Nat Commun* 8**,** 14007. doi: 10.1038/ncomms14007

Kublanov, I.V., Sigalova, O.M., Gavrilov, S.N., Lebedinsky, A.V., Rinke, C., Kovaleva, O., Chernyh, N.A., Ivanova, N., Daum, C., Reddy, T.B.K., Klenk, H.-P., Spring, S., Göker, M., Reva, O.N., Miroshnichenko, M.L., Kyrpides, N.C., Woyke, T., Gelfand, M.S., and Bonch-Osmolovskaya, E.A. (2017). Genomic Analysis of *Caldithrix abyssi*, the Thermophilic Anaerobic Bacterium of the Novel Bacterial Phylum *Calditrichaeota*. *Front Microbiol* 8**,** 195. doi: 10.3389/fmicb.2017.00195

Li, H., Handsaker, B., Wysoker, A., Fennell, T., Ruan, J., Homer, N., Marth, G., Abecasis, G., Durbin, R., and Genome Project Data Processing, S. (2009). The Sequence Alignment/Map format and SAMtools. *Bioinformatics* 25**,** 2078-2079. doi: 10.1093/bioinformatics/btp352

Liu, Y., Wang, Z., Liu, J., Levar, C., Edwards, M.J., Babauta, J.T., Kennedy, D.W., Shi, Z., Beyenal, H., Bond, D.R., Clarke, T.A., Butt, J.N., Richardson, D.J., Rosso, K.M., Zachara, J.M., Fredrickson, J.K., and Shi, L. (2014). A trans-outer membrane porin-cytochrome protein complex for extracellular electron transfer by *Geobacter sulfurreducens* PCA. *Environ Microbiol Rep* 6**,** 776-785. doi: 10.1111/1758-2229.12204

Llirós, M., García-Armisen, T., Darchambeau, F., Morana, C., Triadó-Margarit, X., Inceoğlu, Ö., Borrego, C.M., Bouillon, S., Servais, P., Borges, A.V., Descy, J.-P., Canfield, D.E., and Crowe, S.A. (2015). Pelagic photoferrotrophy and iron cycling in a modern ferruginous basin. *Sci Rep* 5. doi: 10.1038/srep13803

Losey, N.A., Stevenson, B.S., Busse, H.-J., Sinninghé Damste, J.S., Rijpstra, W.I.C., Rudd, S., and Lawson, P.A. (2013). *Thermoanaerobaculum aquaticum* gen. nov., sp. nov., the first cultivated member of *Acidobacteria* subdivision 23, isolated from a hot spring. *Int J Syst Evol Micr* 63**,** 4149-4157. doi: 10.1099/ijs.0.051425-0

Lovley, D. (2006). "Dissimilatory Fe(III)- and Mn(IV)-Reducing Prokaryotes," in *The Prokaryotes,* eds. M. Dworkin, S. Falkow, E. Rosenberg, K.H. Schleifer & E. Stackebrandt. (New York, NY: Springer), 635-658.

Lovley, D.R., Holmes, D.E., and Nevin, K.P. (2004). Dissimilatory Fe(III) and Mn(IV) Reduction. *Adv Microb Physiol* 49**,** 219-286. doi: 10.1016/s0065-2911(04)49005-5

Lücker, S., Wagner, M., Maixner, F., Pelletier, E., Koch, H., Vacherie, B., Rattei, T., Sinninghe Damsté, J.S., Spieck, E., Le Paslier, D., and Daims, H. (2010). A *Nitrospira* metagenome illuminates the physiology and evolution of globally important nitrite-oxidizing bacteria. *Proc Natl Acad Sci USA* 107**,** 13479-13484. doi: 10.1073/pnas.1003860107

Matschiavelli, N., Oelgeschläger, E., Cocchiararo, B., Finke, J., and Rother, M. (2012). Function and Regulation of Isoforms of Carbon Monoxide Dehydrogenase/Acetyl Coenzyme A Synthase in *Methanosarcina Acetivorans*. *J Bacteriol* 194**,** 5377-5387. doi: 10.1128/JB.00881-12

Mavromatis, K., Ivanova, N.N., Chen, I.M., Szeto, E., Markowitz, V.M., and Kyrpides, N.C. (2009). The DOE-JGI Standard Operating Procedure for the Annotations of Microbial Genomes. *Stand Genomic Sci* 1**,** 63-67. doi: 10.4056/sigs.632

Mccleskey, R.B., Nordstrom, D.K., Susong, D.D., Ball, J.W., and Holloway, J.M. (2010). Source and fate of inorganic solutes in the Gibbon River, Yellowstone National Park, Wyoming, USA. *J Volcanol Geoth Res* 193**,** 189-202. doi: 10.1016/j.jvolgeores.2010.03.014

Miroshnichenko, M.L., Kolganova, T.V., Spring, S., Chernyh, N., and Bonch-Osmolovskaya, E.A. (2010). Caldithrix palaeochoryensis sp. nov., a thermophilic, anaerobic, chemo-organotrophic bacterium from a geothermally heated sediment, and emended description of the genus Caldithrix. *Int J Syst Evol Micr* 60**,** 2120-2123. doi: 10.1099/ijs.0.016667-0

Miroshnichenko, M.L., Kostrikina, N.A., Chernyh, N.A., Pimenov, N.V., Tourova, T.P., Antipov, A.N., Spring, S., Stackebrandt, E., and Bonch-Osmolovskaya, E.A. (2003). *Caldithrix abyssi* gen. nov., sp. nov., a nitrate-reducing, thermophilic, anaerobic bacterium isolated from a Mid-Atlantic Ridge hydrothermal vent, represents a novel bacterial lineage. *Int J Syst Evol Micr* 53**,** 323-329. doi: 10.1099/ijs.0.02390-0

Neubauer, S.C., Emerson, D., and Megonigal, J.P. (2002). Life at the Energetic Edge: Kinetics of Circumneutral Iron Oxidation by Lithotrophic Iron-Oxidizing Bacteria Isolated from the Wetland-Plant Rhizosphere. *Appl Environ Microbiol* 68**,** 3988-3995. doi: 10.1128/aem.68.8.3988-3995.2002

Nurk, S., Meleshko, D., Korobeynikov, A., and Pevzner, P. (2016). metaSPAdes: a new versatile de novo metagenomics assembler. *arXiv* q-Bio.GN. doi: arXiv:1604.03071v2

Orcutt, B.N., Sylvan, J.B., Rogers, D.R., Delaney, J., Lee, R.W., and Girguis, P.R. (2015). Carbon fixation by basalt-hosted microbial communities. *Front Microbiol* 6**,** 904. doi: 10.3389/fmicb.2015.00904

Parenteau, M.N., and Cady, S.L. (2010). Microbial Biosignatures in Iron-Mineralized Phototrophic Mats at Chocolate Pots Hot Springs, Yellowstone National Park, United States. *Palaios* 25**,** 97-111. doi: 10.2110/palo.2008.p08-133r

Parenteau, M.N., Jahnke, L.L., Farmer, J.D., and Cady, S.L. (2014). Production and Early Preservation of Lipid Biomarkers in Iron Hot Springs. *Astrobiology* 14**,** 502-521. doi: 10.1089/ast.2013.1122

Parks, D.H., Imelfort, M., Skennerton, C.T., Hugenholtz, P., and Tyson, G.W. (2015). CheckM: assessing the quality of microbial genomes recovered from isolates, single cells, and metagenomes. *Genome Res* 25**,** 1043-1055. doi: 10.1101/gr.186072.114

Pérez-Rodríguez, I., Rawls, M., Coykendall, D.K., and Foustoukos, D.I. (2016). Deferrisoma palaeochoriense sp. nov., a thermophilic, iron(III)-reducing bacterium from a shallow-water hydrothermal vent in the Mediterranean Sea. *Int J Syst Evol Micr* 66**,** 830-836. doi: 10.1099/ijsem.0.000798

Pierson, B.K., and Parenteau, M.N. (2000). Phototrophs in high iron microbial mats: microstructure of mats in iron-depositing hot springs. *FEMS Microbiol Ecol* 32**,** 181-196. doi:

Pierson, B.K., Parenteau, M.N., and Griffin, B.M. (1999). Phototrophs in High-Iron-Concentration Microbial Mats: Physiological Ecology of Phototrophs in an Iron-Depositing Hot Spring. *Appl Environ Microbiol* 65**,** 5474-5483. doi:

Podosokorskaya, O.A., Kadnikov, V.V., Gavrilov, S.N., Mardanov, A.V., Merkel, A.Y., Karnachuk, O.V., Ravin, N.V., Bonch-Osmolovskaya, E.A., and Kublanov, I.V. (2013). Characterization of *Melioribacter roseus* gen. nov., sp. nov., a novel facultatively anaerobic thermophilic cellulolytic bacterium from the class *Ignavibacteria*, and a proposal of a novel bacterial phylum *Ignavibacteriae*. *Environ Microbiol* 15**,** 1759-1771. doi: 10.1111/1462-2920.12067

Roden, E.E., Mcbeth, J.M., Blothe, M., Percak-Dennett, E.M., Fleming, E.J., Holyoke, R.R., Luther, G.W., 3rd, Emerson, D., and Schieber, J. (2012). The Microbial Ferrous Wheel in a Neutral pH Groundwater Seep. *Front Microbiol* 3**,** 172. doi: 10.3389/fmicb.2012.00172

Ruff, S.W., and Farmer, J.D. (2016). Silica deposits on Mars with features resembling hot spring biosignatures at El Tatio in Chile. *Nat Commun* 7**,** 13554. doi: 10.1038/ncomms13554

Santos, T.C., Silva, M.A., Morgado, L., Dantas, J.M., and Salgueiro, C.A. (2015). Diving into the redox properties of Geobacter sulfurreducens cytochromes: a model for extracellular electron transfer. *Dalton Trans* 44**,** 9335-9344. doi: 10.1039/c5dt00556f

Say, R.F., and Fuchs, G. (2010). Fructose 1,6-bisphosphate aldolase/phosphatase may be an ancestral gluconeogenic enzyme. *Nature* 464**,** 1077-1081. doi: 10.1038/nature08884

Schauder, R., Preuß, A., Jetten, M., and Fuchs, G. (1989). Oxidative and reductive acetyl CoA/carbon monoxide dehydrogenase pathway in *Desulfohacterium autotrophicum*. *Arch Microbiol* 151**,** 84-89. doi:

Sekiguchi, Y., Muramatsu, M., Imachi, H., Narihiro, T., Ohashi, A., Harada, H., Hanada, S., and Kamagata, Y. (2008). *Thermodesulfovibrio aggregans* sp. nov. and *Thermodesulfovibrio thiophilus* sp. nov., anaerobic, thermophilic, sulfate-reducing bacteria isolated from thermophilic methanogenic sludge, and emended description of the genus *Thermodesulfovibrio*. *Int J Syst Evol Micr* 58**,** 2541-2548. doi: 10.1099/ijs.0.2008/000893-0

Shi, L., Dong, H., Reguera, G., Beyenal, H., Lu, A., Liu, J., Yu, H.-Q., and Fredrickson, J.K. (2016). Extracellular electron transfer mechanisms between microorganisms and minerals. *Nat Rev Microbiol* 14**,** 651-662. doi: 10.1038/nrmicro.2016.93

Shi, L., Fredrickson, J.K., and Zachara, J.M. (2014). Genomic analyses of bacterial porin-cytochrome gene clusters. *Front Microbiol* 5**,** 657. doi: 10.3389/fmicb.2014.00657

Slobodkina, G.B., Reysenbach, A.L., Panteleeva, A.N., Kostrikina, N.A., Wagner, I.D., Bonch-Osmolovskaya, E.A., and Slobodkin, A.I. (2012). *Deferrisoma camini* gen. nov., sp. nov., a moderately thermophilic, dissimilatory iron(III)-reducing bacterium from a deep-sea hydrothermal vent that forms a distinct phylogenetic branch in the *Deltaproteobacteria*. *Int J Syst Evol Micr* 62**,** 2463-2468. doi: 10.1099/ijs.0.038372-0

Squyres, S.W., Arvidson, R.E., Ruff, S., Gellert, R., Morris, R.V., Ming, D.W., Crumpler, L., Farmer, J.D., Des Marais, D.J., Yen, A., Mclennan, S.M., Calvin,

W., Bell, J.F., 3rd, Clark, B.C., Wang, A., Mccoy, T.J., Schmidt, M.E., and De Souza, P.A., Jr. (2008). Detection of Silica-Rich Deposits on Mars. *Science* 320**,** 1063-1067. doi:

Strauss, G., and Fuchs, G. (1993). Enzymes of a novel autotrophic CO_2 fixation pathway in the phototrophic bacterium *Chloroflexus aurantiacus*, the 3-hydroxypropionate cycle. *Eur J Biochem* 215**,** 633-643. doi:

Suzuki, D., Li, Z., Cui, X., Zhang, C., and Katayama, A. (2014). Reclassification of *Desulfobacterium anilini* as *Desulfatiglans anilini* comb. nov. within *Desulfatiglans* gen. nov., and description of a 4-chlorophenol-degrading sulfate-reducing bacterium, *Desulfatiglans parachlorophenolica* sp. nov. *Int J Syst Evol Micr* 64**,** 3081-3086. doi: 10.1099/ijs.0.064360-0

Tabita, F.R., Satagopan, S., Hanson, T.E., Kreel, N.E., and Scott, S.S. (2008). Distinct form I, II, III, and IV Rubisco proteins from the three kingdoms of life provide clues about Rubisco evolution and structure/function relationships. *J Exp Bot* 59**,** 1515-1524. doi: 10.1093/jxb/erm361

Tang, K.-H., Tang, Y.J., and Blankenship, R.E. (2011). Carbon metabolic pathways in phototrophic bacteria and their broader evolutionary implications. *Front Microbiol* 2**,** 165. doi: 10.3389/fmicb.2011.00165

Tank, M., Thiel, V., Ward, D.M., and Bryant, D.A. (2017). "A Panoply of Phototrophs: An Overview of the Thermophilic Chlorophototrophs of the Microbial Mats of Alkaline Siliceous Hot Springs in Yellowstone National Park, WY, USA," in *Modern Topics in the Phototrophic Prokaryotes,* ed. P.C. Hallenbeck. (Switzerland: Springer International), 87-137.

Taylor, S.R., and Mclennan, S.M. (1985). "The continental crust: its composition and evolution." (Oxford: Blackwell Scientific Publications).

Taylor, S.R., and Mclennan, S.M. (2009). "Mars: crustal composition and evolution," in *Planetary Crusts: Their Composition, Origin, and Evolution*. (Cambridge, UK: Cambridge University Press), 141-180.

Trouwborst, R.E., Johnston, A., Koch, G., Luther, G.W., and Pierson, B.K. (2007). Biogeochemistry of Fe(II) oxidation in a photosynthetic microbial mat: Implications for Precambrian Fe(II) oxidation. *Geochim Cosmochim Ac* 71**,** 4629-4643. doi: 10.1016/j.gca.2007.07.018

Van Der Meer, M.T.J., Klatt, C.G., Wood, J., Bryant, D.A., Bateson, M.M., Lammerts, L., Schouten, S., Sinninghe Damsté, J.S., Madigan, M.T., and Ward, D.M. (2010). Cultivation and genomic, nutritional, and lipid biomarker characterization of *Roseiflexus* strains closely related to predominant *in situ* populations inhabiting Yellowstone hot spring microbial mats. *J Bacteriol* 192**,** 3033-3042. doi: 10.1128/JB.01610-09

Vargas, M., Kashefi, K., Blunt-Harris, E.L., and Lovley, D.R. (1998). Microbiological evidence for Fe(III) reduction on early Earth. *Nature* 395**,** 65-67. doi:

Ward, L.M., Idei, A., Terajima, S., Kakegawa, T., Fischer, W.W., and Mcglynn, S.E. (2017). Microbial diversity and iron oxidation at Okuoku-hachikurou Onsen, a Japanese hot spring analog of Precambrian iron formations. *Geobiology* 15**,** 817-835. doi: 10.1111/gbi.12266

Weiss, J.V., Rentz, J.A., Plaia, T., Neubauer, S.C., Merrill-Floyd, M., Lilburn, T., Bradburne, C., Megonigal, J.P., and Emerson, D. (2007). Characterization of

Neutrophilic Fe(II)-Oxidizing Bacteria Isolated from the Rhizosphere of Wetland Plants and Description of *Ferritrophicum radicicolagen*. nov. sp. nov., and *Sideroxydans paludicola* sp. nov. *Geomicrobiol J* 24**,** 559-570. doi: 10.1080/01490450701670152

Williams, T.J., Zhang, C.L., Scott, J.H., and Bazylinski, D.A. (2006). Evidence for Autotrophy via the Reverse Tricarboxylic Acid Cycle in the Marine Magnetotactic Coccus Strain MC-1. *Appl Environ Microbiol* 72**,** 1322-1329. doi: 10.1128/AEM.72.2.1322-1329.2006

Zaharia, M., Bolonsky, W.J., Curtis, K., Fox, A., Patterson, D., Shenker, S., Stoica, I., Karp, R.M., and Sittler, T. (2011). Faster and More Accurate Sequence Alignment with SNAP. *arXiv* cs.DS. doi: arXiv:1111.5572v1

Zarzycki, J., Brecht, V., Müller, M., and Fuchs, G. (2009). Identifying the missing steps of the autotrophic 3-hydroxypropionate CO_2 fixation cycle in *Chloroflexus aurantiacus*. *Proc Natl Acad Sci USA* 106**,** 21317-21322. doi: 10.1073/pnas.0908356106

Zarzycki, J., Schlichting, A., Strychalsky, N., Müller, M., Alber, B.E., and Fuchs, G. (2008). Mesaconyl-coenzyme A hydratase, a new enzyme of two central carbon metabolic pathways in bacteria. *J Bacteriol* 190**,** 1366-1374. doi: 10.1128/JB.01621-07

Zhang, H., Sekiguchi, Y., Hanada, S., Hugenholtz, P., Kim, H., Kamagata, Y., and Nakamura, K. (2003). *Gemmatimonas aurantiaca* gen. nov., sp. nov., a gram-negative, aerobic, polyphosphate-accumulating micro-organism, the first cultured representative of the new bacterial phylum *Gemmatimonadetes* phyl. nov. *Int J Syst Evol Micr* 53**,** 1155-1163. doi: 10.1099/ijs.0.02520-0
