# Supplementary material for: Investigating the Composition and Metabolic Potential of Microbial Communities in Chocolate Pots Hot Springs
Source: Front Microbiol. 2018 Sep 7;9:2075. doi: 10.3389/fmicb.2018.02075 (PMC6137239; doi:10.3389/fmicb.2018.02075)
Supplement: Supplementary file 9 [file Image_1.PDF]

2. Supplementary Figures

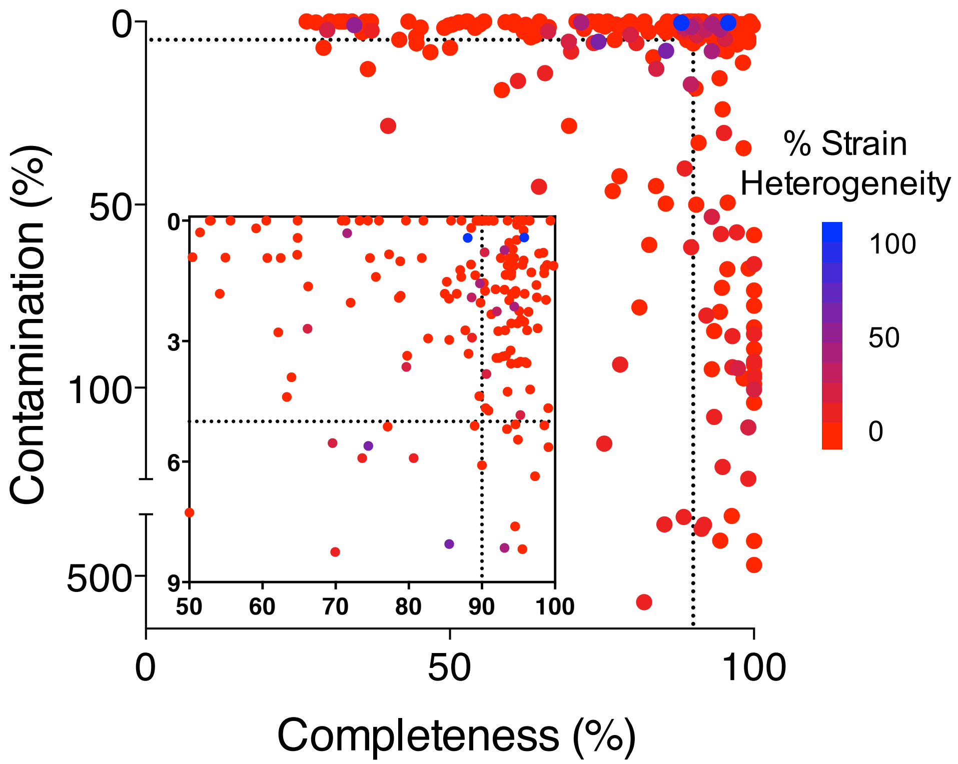

Supplementary Figure 1. Completeness, contamination, and strain heterogeneity calculated using CheckM for each MAG binned in CONCOCT for the CP core metagenomic co-assembly, following manual splitting of composite bins, and before removal of partially complete and very contaminated MAGs. Inset panel shows the MAGs with greater than 50% completeness and less than 10% contamination. The "contamination" statistic calculated in CheckM is a measurement of redundant marker and the additional measurement of "strain heterogeneity" is used to determine the similarity of the phylogeny of the redundant marker genes. MAGs with high contamination, along with high strain heterogeneity, represent very closely related organisms that the binning algorithm was unable to parse out (e.g. strains of the same species).
