# Supplementary material for: Investigating the Composition and Metabolic Potential of Microbial Communities in Chocolate Pots Hot Springs
Source: Front Microbiol. 2018 Sep 7;9:2075. doi: 10.3389/fmicb.2018.02075 (PMC6137239; doi:10.3389/fmicb.2018.02075)
Supplement: Supplementary file 10 [file Image_2.PDF]

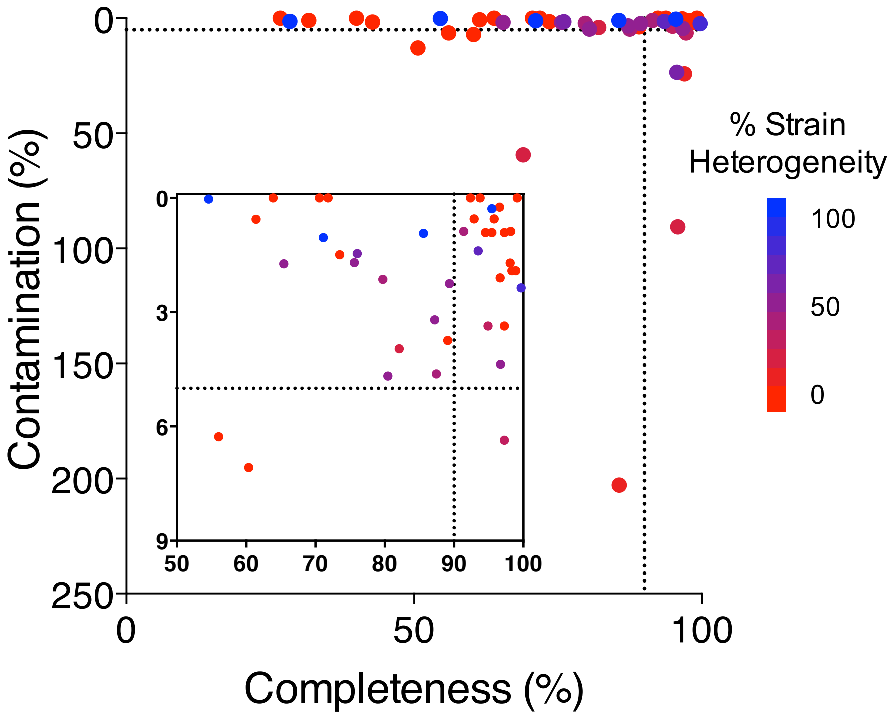

Supplementary Figure 2. Completeness, contamination and strain heterogeneity calculated using CheckM for each MAG clustered using CONCOCT for the CP vent pool metagenomic assembly. Inset panel shows the MAGs with greater than 50% completeness and less than 10% contamination.
