# Supplementary material for: Investigating the Composition and Metabolic Potential of Microbial Communities in Chocolate Pots Hot Springs
Source: Front Microbiol. 2018 Sep 7;9:2075. doi: 10.3389/fmicb.2018.02075 (PMC6137239; doi:10.3389/fmicb.2018.02075)
Supplement: Supplementary file 11 [file Image_3.PDF]

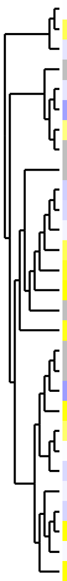

Core 1	Core 2	Core 3	MAG #	% Completeness	*Geobacter*-like *pcc*	*pcc*-like	*mto*-like	CBB	TAS	WL	rTCA	3HP	Concensus Phylogeny
	**Normalized Coverage**					**Putative EET System**				**Putative CO_2 fixation Pathway**			
11.10	159.47		**1**	97.47									Euryarchaeota
3.85	45.98	1.05	**164.2**	94.01				-					*Nitrosopumilaceae*
25.16			**14**	89.72					o				Archaea
	27.49	1.04	**245.3**	92.59				-					*Chthonomonas calidirosea*
24.92			**70**	82.00						+	-		*Anaerolinea thermophila*
65.37	11.55		**165**	71.59						+			Chloroflexi
5.36	38.35	2.39	**109**	95.71								-	*Dehalococcoides*
	2.69	14.10	**234.2**	93.73								o	*Dehalococcoides*
		148.24	**197.1**	95.54	-	+		-					*Caldithrix abyssi*
37.62	7.89		**220.3**	88.51	o	-							*Ignavibacteriales*
32.57	2.20		**220.2**	93.44	o	-							*Ignavibacteriales*
16.14	3.82		**79.2**	95.34	+						-		*Ignavibacteriales*
3.86	16.74	117.50	**39**	96.17	+	-					-		*Ignavibacteriales*
2.05	81.33	4.40	**34**	93.97	+								*Ignavibacteriales*
1.07	6.01	13.72	**174**	97.81	+						-		*Ignavibacteriales*
	21.99	64.97	**209**	93.72	o								*Ignavibacteriales*
1.88	34.07	13.23	**251**	98.90			+				-		*Gemmatimonas aurantiaca*
		14.11	**4**	94.94		+					-		*Ca.* Nitrospira defluvii
	45.99		**250.2**	95.85							+		*Ca.* Nitrospira defluvii
67.66	8.83	1.26	**64.4**	90.91			+			+			*Thermodesulfovibrio*
1.38	8.68		**227**	93.97			+	+			-		*Thermodesulfovibrio*
4.61	31.94		**69.1**	94.87		+							*Acidobacteriaceae*
11.47	3.96		**236**	94.44		++							Acidobacteria
32.15			**235**	95.73		++					o		Acidobacteria
17.58			**64.2**	94.84		+				+	-		*Desulfobacterium anilini*
34.84	3.21		**69.2**	95.80	++						-		*Deferrisoma camini*
1.10	4.34	30.57	**0**	96.59							o		*Deferrisoma camini*
14.33	1.03		**68**	95.80	+					+	-		*Deferrisoma camini*
1.07	59.75	13.43	**171**	95.24				+					*Sideroxydans lithotrophicus*

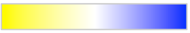

Supplementary Figure 3. The twenty highest coverage MAGs from the entire CP core co-assembly, and abundant MAGs from individual cores that encoded putative metabolic processes of interest, e.g. EET and CO_2 fixation. Metagenomic reads from individual core samples were mapped against the co-assembly to visualize how the abundance of certain MAGs changes with distance from the vent source. High-coverage (abundant) MAGs are highlighted in blue, low-coverage MAGs are in yellow, and MAGs that were below detection in a core sample (average coverage of mapped reads <1.0) are grayed out. Percent completeness of each MAG was calculated based on the presence of single-copy marker genes in CheckM. Putative phylogenetic identity of MAGs was determined by a consensus between CheckM and BLAST/MEGAN. The phylogenetic tree was produced in Dendroscope using output from CheckM. Putative EET systems were positively detected (green +) in MAGs encoding an OM porin, associated *c*-cyts, and all supplemental genes. MAGs that code for multiple sets of EET genes are indicated (green ++). MAGs encoding a homolog to the *Geobacter*-like *pcc* porin, and if no more than one of the predicted associated *c*-cyts was undetected, were considered partially complete (purple o). MAGs encoding an incomplete set of genes were considered too incomplete for further analysis (red -). For carbon fixation, MAGs that encoded a complete set of genes involved in the CBB, WL, or rTCA pathways are indicated (green +). MAGs that encoded all key marker genes, and if no more than one of the additional genes predicted for a given pathway was undetected, were classified as partially complete, and were considered to be potentially involved in CO_2 fixation (purple o). MAGs that did not encode any key marker genes were considered too incomplete for further analysis (red -). Remaining MAGs were abundant in the metagenomic co-assembly but had no obvious involvement in Fe transformation or carbon fixation. Abbreviation: *Ca.*, *Candidatus*; CBB, Calvin-Benson-Bassham cycle; TAS, thiazole adenylate synthase; WL, Wood-Ljungdahl pathway; rTCA, reductive tricarboxylic acid cycle; 3HP, 3-hydroxypropionate cycle.
