# Supplementary material for: Investigating the Composition and Metabolic Potential of Microbial Communities in Chocolate Pots Hot Springs
Source: Front Microbiol. 2018 Sep 7;9:2075. doi: 10.3389/fmicb.2018.02075 (PMC6137239; doi:10.3389/fmicb.2018.02075)
Supplement: Supplementary file 12 [file Image_4.PDF]

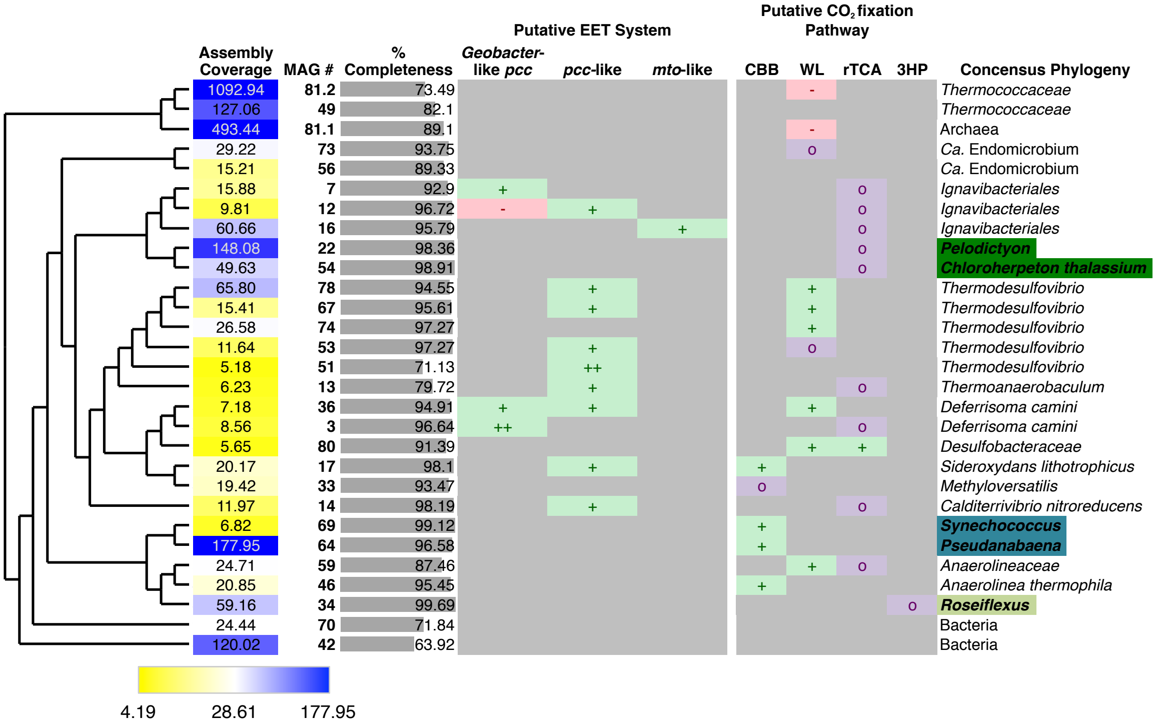

Supplementary Figure 4. The Eleven above-average coverage (28.61) MAGs from the CP vent pool assembly, and all MAGs containing putative metabolic processes of interest. High-coverage (abundant) MAGs are highlighted in blue and low-coverage bins are in yellow. The percent completeness of each MAG was calculated based on the presence of single-copy marker genes in CheckM. Putative phylogenetic identity of MAGs was determined by a consensus between CheckM and BLAST/MEGAN. The phylogenetic tree was produced in Dendroscope using output from CheckM. Putative EET systems were positively detected (green +) in MAGs containing an OM porin, associated *c*-cyts, and all supplemental genes. MAGs encoding multiple sets of EET genes are indicated (green ++). MAGs encoding an incomplete set of genes were considered too incomplete for further analysis (red -). MAGs that encoded a complete set of genes involved in the CBB or WL pathways are indicated (green +). MAGs encoding all key marker genes, and if no more than one of the additional genes predicted for a given pathway was undetected, were classified as partially complete and were considered to be potentially involved in CO_2 fixation (purple o). MAGs that did not encode any key marker genes were considered too incomplete for further analysis (red -). Putative photosynthetic MAGs are highlighted to indicate oxygenic (cyan) and anoxygenic (green sulfur bacteria, dark green; green non-sulfur bacteria, light green) phototrophs. The remaining MAGs were abundant in the metagenomic assembly but had no obvious involvement in Fe transformation or carbon fixation. Abbreviation: *Ca.*, *Candidatus*; CBB, Calvin-Benson-Bassham cycle; WL, Wood-Ljungdahl pathway; rTCA, reductive tricarboxylic acid cycle; 3HP, 3-hydroxypropionate cycle.
